# Nanostructured Ti–6Al–4V Reduces Adhesion of Several Bacterial Species: An In Vitro Study

**DOI:** 10.1002/smsc.70348

**Published:** 2026-07-28

**Authors:** Sadaf Khalatbarizamanpoor, Adrian G. Nowotnick, Stephanie Lippmann, Antje Häder, Felix Otto, Lena Raupach, Torsten Fritz, Yasmina Reisser, Jörg Bossert, Bettina Löffler, Klaus D. Jandt

**Affiliations:** ^1^ Institute of Medical Microbiology Jena University Hospital Jena Germany; ^2^ Excellence Graduate School “Jena School für Microbial Communication (JSMC)” Friedrich Schiller University Jena Jena Germany; ^3^ Otto Schott Institute of Materials Research Friedrich Schiller University Jena Jena Germany; ^4^ Institute of Applied Physics Friedrich Schiller University Jena Jena Germany; ^5^ Institute of Solid State Physics Friedrich Schiller University Jena Jena Germany

**Keywords:** antimicrobial, *Escherichia coli*, nanostructured titanium alloys, physical action, *Staphylococcus aureus*, *Staphylococcus epidermidis*, Ti–6Al–4V

## Abstract

Biomaterial‐associated infections (BAIs) and insufficient early cellular response remain critical challenges for orthopedic implants. We introduce a comprehensive study that bridges current knowledge gaps by examining an early‐stage antimicrobial effect on the clinically relevant alloy Ti–6Al–4V. It combines pathogenic strains with parallel osteoblast assays and tilted‐view SEM analysis to obtain a qualitative understanding of the adhesion mechanisms. Detailed physicochemical characterization revealed a progressive increase in nanoscale roughness and oxide layer thickness, accompanied by selective Al/V depletion and pronounced hydrophilization. To evaluate biological responses, we used standardized in vitro models with *Staphylococcus aureus*, *Staphylococcus epidermidis*, and *Escherichia coli*. Bacterial adhesion was quantified by SYTO9 staining, a GFP‐expressing strain as a viability control, and SEM imaging. Nanostructured (*R*_q_ ≤ 40 nm) surfaces significantly reduced early bacterial attachment compared to polished nanoflat controls. In parallel, osteoblast‐like SaOs‐2 cells showed stable adhesion and spreading, confirmed by phalloidin/DAPI staining and LDH cytotoxicity assay. Together, these results demonstrate that NaOH‐etched Ti–6Al–4V surfaces can impair early microbial adhesion based on physical action and preserve osteoblast compatibility. By integrating advanced materials characterization with microbiological and cell biological assays, we provide a framework for topography‐driven surface design toward infection‐resistant orthopedic implants that support favorable early osteoblast–surface interactions.

## Graphical abstract

Alkaline etching transforms Ti–6Al–4V into nanostructured, highly hydrophilic surfaces with thicker oxide layers and selective alloy depletion. These physicochemical changes reduce early bacterial adhesion across multiple species by limiting effective nanoscale contact points, while osteoblast‐like cells retain good attachment and spreading. Distinct, species‐specific adhesion strategies, even within the same Gram type, underline the need for tailored surface design.

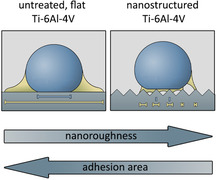

## 1 Introduction

Ti–6Al–4V is the material of choice for load‐bearing orthopedic implants because of its excellent biocompatibility, corrosion resistance, mechanical stability, and long‐term clinical success [1, 2]. It is widely used in total hip and knee arthroplasty as well as in osteosynthesis devices such as plates, screws, and intramedullary nails [3, 5]. Across OECD countries, hip and knee arthroplasty rates continue to rise, and future procedure numbers are expected to increase substantially [6, 7]. Despite this clinical success, biomaterial‐associated infections (BAIs) remain one of the most serious complications in orthopedic surgery [8, 10]. The predominant pathogens are *Staphylococcus aureus* (*S. aureus*) and coagulase‐negative staphylococci, particularly *Staphylococcus epidermidis* (*S. epidermidis*), followed by Gram‐negative bacteria such as *Escherichia coli* (*E. coli*) [11]. Bacterial survival strategies, including adhesin‐mediated attachment, host‐cell invasion, biofilm formation, and phenotype switching, complicate infection treatment and can ultimately lead to implant loosening, tissue destruction, or systemic complications [12, 15]. In parallel, successful implant integration requires rapid osteoblast attachment and osseointegration [16, 17]. However, the biological requirements for mammalian cells and bacteria differ substantially. Osteoblasts interact dynamically with implant surfaces via focal adhesions, whereas bacteria adhere through distinct cell wall‐associated mechanisms and physicochemical interactions [18, 21]. This competition for implant surface colonization is commonly referred to as the “race for the surface” [22]. Consequently, implant surface design must simultaneously promote osteoblast activity while limiting bacterial adhesion [23, 24]. While microscale roughening improves bone integration [25], it may also provide favorable niches for bacterial attachment. Nanoscale surface modifications therefore emerged as a promising strategy to selectively reduce bacterial adhesion while maintaining cellular compatibility [23, 26, 27]. Bioinspired‐nanopatterned systems demonstrated that nanoscale architectures can influence bacterial viability through purely physical interactions [28, 30]. Within titanium systems, approaches such as TiO_2_ thin films and alkaline hydrothermal treatments showed antibacterial potential [31, 33]. However, direct comparison between studies remains difficult because of differences in material systems, surface morphology, and chemistry [30].

Previous studies on nanostructured titanium‐based surfaces remain fragmented across materials, bacterial species, and methodological approaches. Many investigations focused on commercially pure titanium (cp‐Ti), although Ti–6Al–4V represents the clinically dominant alloy for load‐bearing implants [31, 33, 39]. Since alloy composition influences oxide formation and nanoscale surface morphology, findings obtained on cp‐Ti cannot necessarily be transferred directly to Ti–6Al–4V [40, 41]. In addition, most studies focused primarily on *S. aureus*, whereas *S. epidermidis* and Gram‐negative species such as *E. coli* were investigated less frequently despite their clinical relevance [42, 43]. Furthermore, antibacterial effects observed after chemical etching are often difficult to attribute exclusively to nanotopography because possible chemical contributions from the etching process are insufficiently characterized. In the present study, we established a controlled NaOH‐etched Ti–6Al–4V nanoroughness series (*R*_q_ ≈ 10–40 nm), bridging the intermediate range between smoother nanoscale films and more pronounced nanostructured systems [31, 33, 40, 41, 44]. Detailed physicochemical surface characterization was performed to distinguish topographical effects from possible chemical influences. Early bacterial adhesion of *S. aureus*, *S. epidermidis*, and *E. coli* was evaluated after 1 and 24 h using fluorescence‐based assays and scanning electron microscopy (SEM). These time points were specifically selected because the initial adhesion phase is considered a critical determinant of subsequent bacterial colonization and infection development on implant surfaces. In parallel, osteoblast compatibility was assessed using SaOs‐2 cells by LDH cytotoxicity assay and fluorescence staining. We hypothesized that defined nanostructured Ti–6Al–4V surfaces reduce early bacterial colonization while maintaining osteoblast adhesion and viability.

## 2 Materials and Methods

### 2.1 Ti–6Al–4V Preparation and Etching

Thermally annealed Ti–6Al–4V ELI (Extra Low Interstitials, Grade 23) rods (S + D METALS GmbH, Stelle, Germany, ISO 5832‐3) were used to prepare circular disks with a diameter of 14 mm and a thickness of approximately 2 mm. The rods were sectioned using a low‐speed diamond saw (Isomet 5000, HC15, Buehler, Lake Bluff, IL, USA) under continuous water cooling to prevent thermal alteration. The disks were then ground under running water in nine successive steps using diamond‐embedded abrasive papers with particle sizes decreasing from 165 to 0.5 µm on a polishing system (Phoenix 4000, Buehler, Lake Bluff, IL, USA), ensuring progressive removal of surface irregularities and a reproducible finish. Final polishing was likewise performed on the same system using a nondrying colloidal silica suspension (OP‐S NonDry, Struers, Willich, Germany; particle size ≈0.25 µm). The polished surfaces were examined by light microscopy (Leica DMXRE, 40×, Wetzlar, Germany) to confirm the absence of scratches or optical irregularities. The samples were placed in a glass vessel and subjected to ultrasonic cleaning in ethanol (96%) and subsequently in double‐distilled water for 10 min each at room temperature (RT). The samples were then mounted on a Teflon holder suspended in a beaker, which was immersed in a larger water‐filled vessel serving as a thermostated water bath (50 °C) with heating and stirring capabilities; the temperature was automatically regulated within ±5 °C.

In accordance with protocols described by Komasa et al. [45, 46], etching was performed using a 10 mol/L sodium hydroxide solution prepared from NaOH pellets (≥99%, Sigma–Aldrich, St. Louis, MO, USA; Product No. 1.06498), resulting in a calculated pH of 15, assuming complete dissociation. Based on optimization, etching was conducted at 50 °C with continuous stirring at 220 rpm. Etching durations were 5 min and 24 h.

Following etching, samples were immediately rinsed twice with double‐distilled water and once with ethanol, immersed for 10 min under constant stirring in double‐distilled water, and subsequently subjected to ultrasonic treatment for 10 min each in ethanol and double‐distilled water. Samples were finally dried using filtered compressed air. Nonetched samples served as controls.

### 2.2 Ti–6Al–4V Surface Characterization

Atomic force microscopy (AFM) was performed using a JPK NanoWizard 4 system equipped with PPP‐RT‐NCHR tips (nominal spring constant 42 N/m, resonance frequency 330 kHz, NANOSENSORS) operated in QI mode to characterize surface topography. Based on the findings of Lüdecke et al. [31], a scan area of 1 × 1 µm was selected with a resolution of 256 × 256 pixels. For each sample, three regions were analyzed at RT in air.

Image processing and analysis were performed with a custom Python 3.11 (Python Software Foundation, Beaverton, OR, USA) script. Plane image leveling, median line correction, and horizontal scar removal were applied to the raw data prior to analysis. The *R*_a_, *R*_q_, and *R*_sk_ were calculated for each pixel row and column of every image, while the remaining parameters (*R*_sa_, peak‐density, peak‐to‐peak distance [PtP], PtV) were derived globally from the full two‐dimensional height maps. Surface gradients were used to calculate *R*_sa_, and peak detection was performed using scipy.signal.find_peaks. Surface roughness parameters (*R*_a_, *R*_q_, *R*_sk_, *R*_sa_) were calculated in accordance with ISO 4287‐1997. *R*_a_ describes the arithmetic mean height deviation from the mean line, whereas *R*_q_ represents the root mean square roughness and is therefore more sensitive to pronounced height variations. *R*_sk_ describes the skewness of the height distribution and indicates whether the surface is dominated by peaks or valleys. *R*_sa_ quantifies the relative increase in true surface area compared with the projected area. Peak density describes the number of detected local maxima per unit area. PtP refers to the lateral peak‐to‐peak spacing between neighboring nanoscale adhesion points, whereas PtV describes the vertical peak‐to‐valley height of the surface features.

Dynamic water contact angle (WCA) measurements were conducted at RT using an *OCA 15EC* (Dataphysics, Filderstadt, Germany) with Milli‐Q water. Immediately prior to measurement, the samples were cleaned as described in Section 2.1. A 2 µL droplet was applied. Due to the rapid wetting behavior of the etched surfaces, stable static contact angles could not be reliably determined. Therefore, the first seconds after droplet deposition were recorded and evaluated as dynamic WCA curves. The contact angle was determined from the average of the left and right contact angles obtained by elliptical fitting. Initial invalid values were excluded, and the first valid contact angle value was defined as *t* = 0 s for each measurement. The dynamic WCA data are provided in the Supporting Information.

### 2.3 Elemental Composition

To obtain complementary information on the elemental distribution in surface and near‐surface regions, a combination of Auger electron spectroscopy (AES), transmission electron microscopy with energy‐dispersive–X‐ray spectroscopy (TEM–EDX), and SEM with EDX (SEM–EDX) was employed.

For TEM–EDX measurements, a protective platinum layer was first deposited onto the surface via electron beam‐induced deposition (EBID), followed by focused ion beam (FIB) milling using a ZEISS FIB‐SEM Auriga 60 (Carl Zeiss, Oberkochen, Germany) with Schottky emitter to create a lamella protruding from an unetched Ti–6Al–4V sample. The final lamella thickness was approximately 100 nm. The lamella was mounted on a sample holder and investigated in bright‐field mode using a transmission electron microscope JEOL NEOARM 200F equipped with a cold field emission gun (JEOL Ltd., Tokyo, Japan). The lateral elemental distributions across the oxide layer were measured by TEM–EDX linescan. The errors were determined using standard deviation.

AES depth profiling was performed using a commercial setup purchased from Varian Inc. (Palo Alto, CA, USA) with an electron acceleration energy of 5.0 keV at an angle of incidence of 30° under ultra‐high vacuum conditions (base pressure in 10^−10^ mbar range). Sputtering was carried out using Kr at 2.0 keV. The elemental depth profiles were recorded as a function of sputter time and converted to depth using the known sputtering rate. Oxygen profiles were modeled using a convolution of a Gaussian and a Heaviside (step) function. Layer thicknesses were extracted from the fitted curves as full‐width‐at‐half‐maximum (FWHM) for the respective oxide and transition regions. Associated uncertainties were calculated via standard Gaussian error propagation.

SEM–EDX measurements were carried out using a ZEISS FIB–SEM Auriga 60 equipped with an EDX detector (Oxford Instruments, Abingdon, UK; X‐Max 80). Spectra were acquired over a surface area of 1050 µm^2^ using primary beam energies of 5, 10, and 15 keV. Quantification was performed with the AZtec software (Oxford Instruments, Abingdon, UK) using standardless P/B‐ZAF matrix correction.

### 2.4 Preparation of Bacterial Infection Inoculum

To ensure consistent bacterial concentrations across experimental conditions, bacterial strains were standardized to an optical density (OD) of 1 at 578 nm during the exponential growth phase. A single colony from each strain was inoculated into 10 mL of brain heart infusion (BHI) medium (Oxoid, Thermo Fisher Scientific, Waltham, USA) and incubated at 37 °C with continuous shaking at 160 rpm for 15–18 h. The overnight culture was then diluted to an OD of 0.05 at 578 nm (measured using a Shimadzu UV‐1202 photometer) in 30 mL of fresh BHI and further incubated under the same conditions for 3 h to reach the log growth phase.

After incubation, bacterial cells were pelleted by centrifugation at 5 000 rpm for 10 min at 4 °C. The supernatant was discarded, and the pellet was resuspended in 10 mL of phosphate‐buffered saline (PBS) (Gibco, Thermo Fisher Scientific, Waltham, USA), followed by another centrifugation and supernatant removal, as described above. This washing step was repeated twice. The final bacterial suspension was prepared in 1 mL of PBS and adjusted to an OD of 1 at 578 nm. The bacterial suspensions were then aliquoted and stored at –80 °C for future use.

For colony‐forming unit (CFU) quantification, serial dilutions were prepared from an aliquot 1 day before each experiment and plated onto blood agar plates. After 24 h of incubation at 37 °C, CFUs were enumerated using a ColonyQuant HD system (Schuett‐biotec GmbH, Göttingen, Mannheim, Germany).

### 2.5 Ti–6Al–4V Sample Infection

To disinfect the samples, they were immersed in 70% ethanol for 15 min at RT. Following disinfection, ethanol was removed, and the samples were rinsed thoroughly with ultrapure water (Chemiekontor, Mannheim, Germany). Each sample was incubated in 1 mL of BHI medium containing 10^7^ CFU/mL of the respective bacterial strain. Incubation was carried out at 37 °C, 5% CO_2_, and 95% relative humidity for either 1 h or 24 h. These parameters were chosen to maintain physiologically relevant incubation conditions and to ensure consistency with the osteoblast cell culture experiments performed in parallel. Importantly, incubation was not performed under anaerobic conditions, as atmospheric oxygen remained present throughout the experiments. At each time point, the culture medium was carefully removed, and the samples were thoroughly washed with ultrapure water to eliminate nonadherent bacteria.

### 2.6 Fluorescent Staining and Microscopy

The SYTO 9 Green Fluorescent Nucleic Acid Stain (Thermo Fisher Scientific, Waltham, USA) staining solution was prepared according to the manufacturer’s protocol. After the final wash in each infection experiment, 100 µL of the staining solution was added to each sample. The samples were incubated at RT in the dark for 10 min. Following incubation, the samples were gently washed with three times ultrapure water and left to air dry in the dark at RT for 1 h.

To investigate the effect of nanorough surface topographies on the early stages of bacterial adhesion more precisely, a GFP‐expressing *S. aureus* USA300 strain was used. This approach had two main advantages. It eliminated variability introduced by postincubation staining and it also ensured that only metabolically active, viable bacteria were visualized, as GFP expression depends on active bacterial metabolism. The tests with the GFP strains were conducted using the same procedure as previously described.

Imaging was then performed using a fluorescence microscope (Keyence BZ‐X810, Osaka, Japan). For each sample, 266 fluorescence images were acquired by rastering across the sample surface, corresponding to a total scanned area of 7.3 × 7.3 mm.

Negative unetched material controls were included in parallel in every experiment by incubating surfaces with medium alone (without bacteria) under otherwise identical conditions. This control also served to monitor potential media contamination. As these controls consistently showed no signal, they were subsequently excluded from the presented figures.

### 2.7 Statistical Analysis

To standardize data quality and reduce bias, the 20 most homogeneous and in‐focus images per sample were automatically selected prior to quantification. Selection was performed by custom Python scripts that combined metrics of local and global inhomogeneity with Laplacian variance into a weighted quality score. Analyses used the complete dataset. The selected images were aggregated for every respective sample to yield one representative data point per sample. No data points were excluded as outliers, preserving the full spread of the measurements. Each condition included *n* = 6 samples, consisting of two technical replicates from three independent biological experiments. Statistical analysis was performed using the Kruskal–Wallis test. Statistical significance was assessed at *α* = 0.05 using Dunn’s post hoc test with Holm–Šidák correction.

### 2.8 SEM, FIB Milling, and Preparation of Bacterial or SaOs‐2 Cells Samples

Surface morphology of the Ti–6Al–4V samples was initially characterized by a ZEISS FIB‐SEM Auriga 60 SEM equipped with a Gallium liquid metal ion source. Rectangular cross‐section trenches were milled into the surface using 30 kV and 50 pA Ga^3+^ ions. For imaging, the specimens were tilted by 54° relative to the electron beam, and all images were subsequently corrected for the tilt angle. During ion milling, the electron beam was paused to minimize unwanted beam‐induced material redeposition.

For subsequent bacterial imaging, samples were incubated with a defined bacterial suspension for 1, and 24 h, respectively. Following incubation, nonadherent bacteria were removed by rinsing three times with ultrapure water. Fixation was performed in 2.5% glutaraldehyde in PBS (pH 7.4) for 2 h at RT. After fixation, glutaraldehyde was aspirated, and samples were washed twice with ultrapure water for 10 min each. Dehydration was carried out in a graded ethanol series (30%, 50%, 70%, 80% for 10 min each; 90% and 100% for 30 min each).

Critical‐point drying was performed using a K850 device (Quorum Technologies, Laughton, UK). Prepared samples were examined in the uncoated state at the lowest possible acceleration voltage to reduce surface charging. Tilted imaging at 80° relative to the surface normal was used to gain more detailed insight into the bacterium–surface interface.

The same procedure was applied for the SaOs‐2 cells. In this case, 2.5 × 10^5^ cells/mL were incubated on the Ti–6Al–4V samples for 24 h, respectively. After incubation, the cells were fixed and dehydrated following the same protocol as described above, including critical‐point drying.

### 2.9 Fluorescence Staining, Imaging, and Cytotoxicity Assay for SaOs‐2 Cells on Ti–6Al–4V Surfaces

To investigate the effect of surface roughness on cell growth, SaOs‐2 cells at a density of 2.5 × 10^5^ cells/mL were seeded on Ti–6Al–4V samples and incubated for 24 h in Advanced DMEM (Cat. 12491015, Thermo Fisher Scientific, Waltham, USA) supplemented by 5% fetal bovine serum albumin (BSA) and 1% penicillin/streptomycin. Following incubation, the supernatant collected for LDH assay, and cells were fixed with 4% paraformaldehyde in PBS for 10 min at RT. Permeabilization was carried out using 0.1% Triton X‐100 in PBS for 10 min. To minimize nonspecific background staining, a blocking step with 1% BSA in PBS was performed for 10 min.

For visualization of the actin cytoskeleton, samples were incubated with Alexa Fluor 647–conjugated phalloidin (Cat. A30107, Thermo Fisher Scientific, Waltham, USA; stock prepared according to the manufacturer’s instructions, diluted 1:400 in PBS, ∼16.5 nM final concentration) for 15 min at RT in the dark. Nuclei were counterstained using the DAPI Staining Solution (Cat. 130‐111‐570, Miltenyi Biotec, Bergisch Gladbach, Germany; supplied at 10 µg/mL, used at a final concentration of ∼0.1 µg/mL in PBS) for 5–10 min in the dark. After each staining step, samples were gently rinsed with PBS and prepared for fluorescence imaging.

For testing cytotoxicity of the untreated and etched surfaces, an LDH assay was performed using the CyQUANT LDH Cytotoxicity Kit (Invitrogen, Thermo Fisher Scientific Inc., Waltham, USA). Prior to the assay, a separate set of SaOs‐2 samples grown on glass‐bottom dishes was lysed by adding lysis buffer from the kit and incubating for 45 min at 37 °C. 25 µL of culture supernatant from etched Ti–6Al–4V, untreated Ti–6Al–4V, and glass‐bottom‐dish samples (spontaneous release control) and lysed cells (Maximum LDH control) were mixed with 25 µL of reaction mixture and incubated for 30 min in the dark at RT and 500 rpm. Absorbance was measured at 490 and 680 nm after adding 25 µL of stop solution. For cytotoxicity determination, the background (680 nm) was subtracted from the 490 nm absorbance, and cytotoxicity was calculated using the following formula:

$$\begin{array}{l} \%\text{Cytotoxicity}\\ =\frac{\text{Sample absorbance}\;-\;\text{Spontaneous release absorbance}}{\text{Maximum LDH absorbance}\;-\;\text{Spontaneous release absorbance}}\\ \;\;\times 100 \end{array}$$

## 3 Results and Discussion

### 3.1 Materials Characterization

#### 3.1.1 Surface Morphology

**FIGURE 1:**
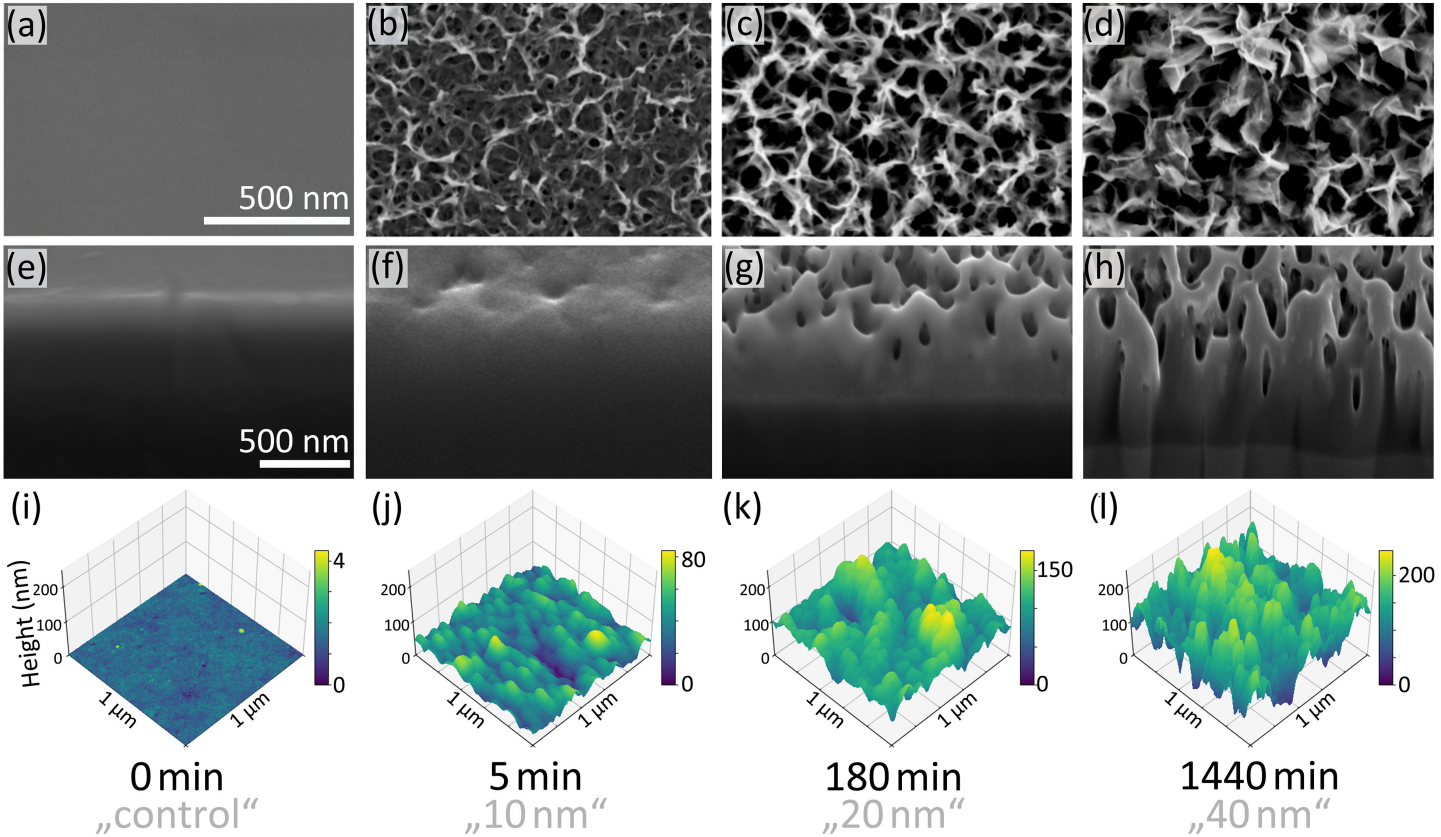
(a–d) SEM images of the four sample groups with increasing surface roughness. (e–h) Respective SEM images of the milled cross‐section trenches. The scale bars in (a) and (e) apply to their respective rows. (i–l) Corresponding AFM topographies of the same groups are shown below with the same z‐scaling. The color scaling is adapted to the respective maximum of each image. The sample groups are later on referred to as control, 10, 20, and 40 nm, based on their approximate *R*_q_ values.

Qualitative analysis of the etched Ti–6Al–4V surfaces revealed a clear dependence of surface morphology on etching duration (Figure 1). SEM images (Figure 1a–h), acquired in secondary electron mode at 2.0 kV acceleration voltage, showed a progressive development of mesh‐like nanostructures with increasing etching time. While the untreated surface appeared smooth and featureless, the etched samples showed increasing pore sizes. The cross‐sections reveal channel‐like patterns beneath the surfaces. The structures become deeper as the roughness increases. The slightly swollen appearance (Figure 1f–h) of the structures is an unavoidable artifact caused by the redeposition of bulk material which was removed during ion beam milling. An estimation of the structural depth based on the cross‐sections is only possible to a very coarse extent. For a more precise characterization of the nanostructured oxide layer, AES measurements are evaluated in the following.

AFM images of topographies (Figure 1i–l) confirmed this trend, displaying a continuous increase in both vertical and lateral surface features. The control sample (0 min) showed minimal topographical variation with a maximum height difference of approximately 5 nm. After 24 h of etching, this height range increased to nearly 300 nm, indicating statistically significant deepening of the surface. Only minor lateral broadening of individual structures was observed, suggesting that surface roughness development was primarily driven by vertical deepening.

All values shown in Table 1 have been determined using Python 3.11, based on NumPy and SciPy libraries [47, 48]. The reported errors correspond to the standard deviations of the image‐level means, reflecting the reproducibility of the respective surface structures after etching.

**TABLE 1:** Surface characterization of etched Ti–6Al–4V samples based on AFM measurements.

Etching time, min	***R***_**a**_ **, nm**	***R***_**q**_ **, nm**	***R***_**sk**_	***R***_**sa**_ **, %**	**Peak density, µm** ^**−2**^	PtP, nm	PtV, nm
0	0.19 ± 0.03	0.24 ± 0.03	0.09 ± 0.23	0.20 ± 0.26	676 ± 45	38 ± 8	4.2 ± 1.6
5	9.4 ± 1.4	11.6 ± 1.7	0.14 ± 0.16	27 ± 16	176 ± 56	75 ± 4	91 ± 17
180	16.0 ± 2.2	19.9 ± 2.6	−0.05 ± 0.11	48 ± 26	149 ± 41	82 ± 3	160 ± 40
1440	29.8 ± 5.5	36.7 ± 6.8	0.02 ± 0.15	86 ± 23	109 ± 18	96 ± 2	290 ± 70

*Note:* Values are reported as mean ± standard deviation (*n* ≥ 5). PtP refers to the lateral distance between neighboring nanoscale peaks, while PtV describes the vertical peak‐to‐valley height of the surface features.

*R*_a_ and *R*_q_ increase with etching time from 0.19 ± 0.03 nm to 29.8 ± 5.5 nm and 0.24 ± 0.03 nm to 36.7 ± 6.8 nm, respectively (Table 1), with all changes being statistically significant. *R*_sk_ values remained close to zero across all samples and showed no clear trend, as their standard deviations were large relative to their means. This indicates an approximately symmetric height distribution. In contrast to the data reported by Lüdecke et al. for physical vapor deposition (PVD) of cp‐Ti [31], the surface area ratio *R*_sa_ increased with surface roughness, from 0.20% ±  0.26% to 86% ± 23%. Peak density decreased with etching, indicating fewer topographic maxima per area. Although etching caused this initial reduction, further changes in peak density with longer durations were minor. The mean horizontal PtP increased from 38 ± 8 to 96 ± 2 nm, and the maximal vertical peak‐to‐valley distance (PtV) rose markedly from 4.2 ± 1.6 to 290 ± 70 nm. PtP was calculated from the peak density, and its uncertainty was derived via Gaussian error propagation. The pronounced increase in PtV confirms that the observed roughness progression is primarily driven by vertical deepening of surface features (rose by a factor of ∼70). The density of topographic maxima decreased markedly after the initial etching step (to approximately one quarter of the control value) and remained essentially constant thereafter. The number of potential nanocontact points available for bacterial attachment, therefore, did not substantially change, whereas their vertical separation from the base plane increased significantly. Here, nanocontact points refer to nanoscale protrusions or raised surface features that can serve as initial physical anchoring sites for bacteria [49].

For clarity, the sample groups are hereafter referred to as control, 10, 20, and 40 nm, based on their approximate *R*_q_ values.

**FIGURE 2:**
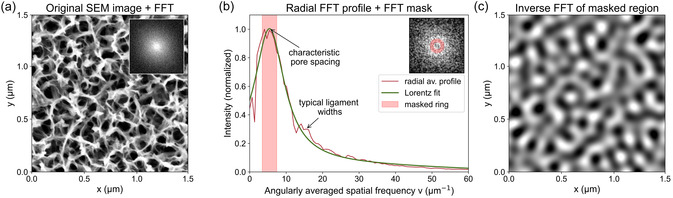
(a) SEM image of the 24 h‐etched surface with corresponding 2D FFT (inset). (b) Radially averaged FFT profile (red) with Lorentz fit (green) and masked frequency band used for ring‐masking in the 2D FFT (inset). (c) Inverse FFT of the masked region highlighting the dominant spatial frequencies.

To further quantify the lateral organization of the etched surfaces, including characteristic pore spacing and ligament width, the SEM images were subjected to a two‐dimensional fast Fourier transform (FFT) analysis (Figure 2). The Hann‐windowed FFT of the 24 h‐etched surface exhibited a circular, ring‐shaped intensity distribution without discernible spots or elliptic distortions, indicating the absence of any preferred in‐plane orientation. The radially averaged FFT magnitude (Figure 2b) was fitted using a skew‐Lorentzian model, yielding a peak spatial frequency of ν_0_ = 5.08 µm^−1^, corresponding to a dominant lateral correlation length of 1/ν_0_ ≈ 0.197 µm, in good agreement with the visually observed characteristic pore‐to‐pore spacing. A broader shoulder at higher frequencies was also observed in the FFT profile, indicating an additional characteristic length scale that corresponds to the average ligament width of approximately 60 nm. Isolating the annular frequency band around ν_0_ (Figure 2b, inset) and reconstructing the corresponding real‐space contribution via inverse FFT (Figure 2c) revealed the recurrent pore–ligament motif at these length scales and confirmed that these structural features are homogeneously distributed across the entire surface. Collectively, these findings corroborate that the etched surfaces form an isotropic nanoscale network governed by statistically dominant pore spacings of ∼200 nm and ligament widths on the order of ∼60 nm.

**FIGURE 3:**
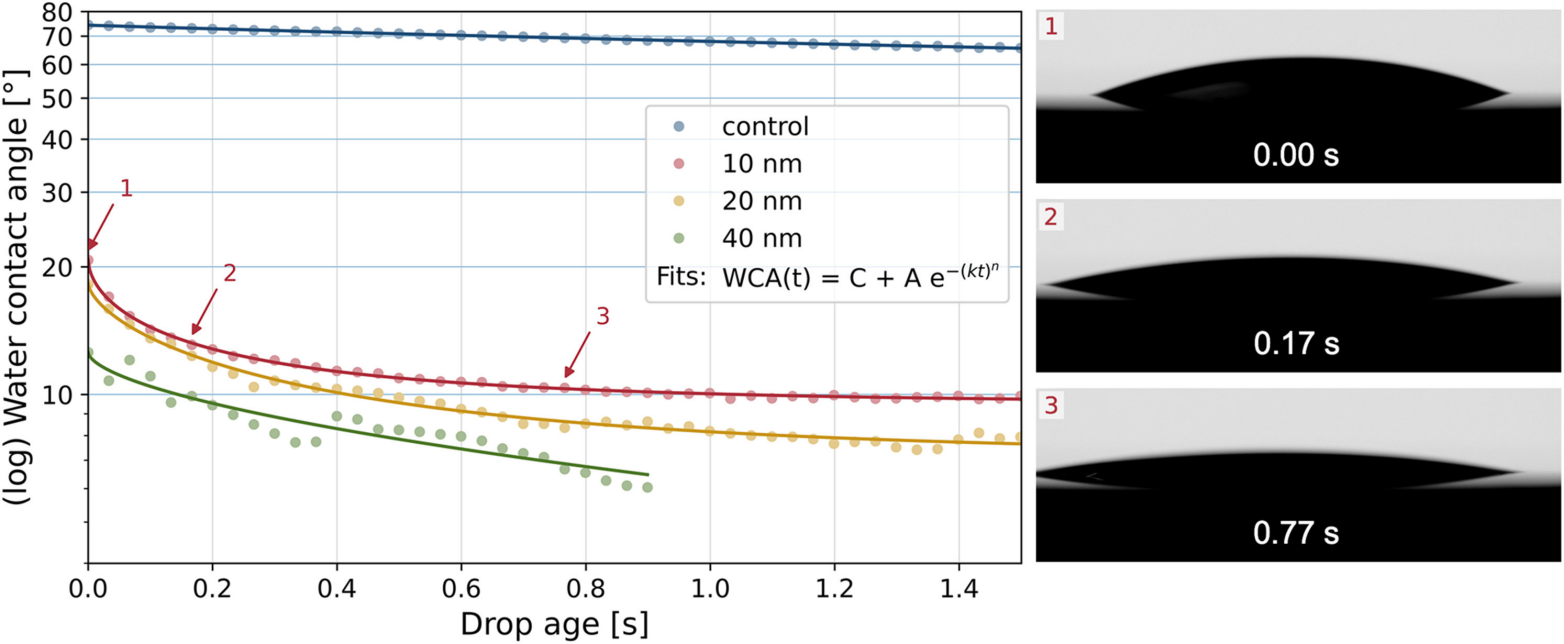
WCA values were plotted over drop age for the polished reference surface (control) and etched surface groups with nominal roughness values of 10, 20, and 40 nm. The arrows indicate the positions of selected video frames for the 10 nm sample (right). The corresponding images illustrate rapid droplet spreading.

Dynamic WCA analysis confirmed a pronounced change in wetting behavior after NaOH etching (Figure 3). Stable static contact angles could not be reliably determined for the etched samples because the droplets spread rapidly and moved beyond the observable region of interest within the first seconds, even at the lowest available optical magnification. Once both droplet edges were no longer fully visible, reliable angle fitting was impossible. Therefore, the first 1.5 s after droplet deposition were analyzed. The polished reference surface exhibited an initial WCA of approximately 75° and showed only a moderate decrease over time, whereas all etched surfaces exhibited substantially faster wetting and markedly lower contact angles, confirming strong hydrophilization after NaOH treatment. The decay for all four sample groups was fitted empirically using a stretched exponential function, $\mathrm{WCA}\left(t\right)=C+Ae^{-{\left(kt\right)}^{n}}$, to compare the nonlinear wetting behavior.

This observation is consistent with previous reports on NaOH‐treated Ti‐6Al‐4V, where contact angles decreased to very low values after alkaline surface modification. Luo et al. reported a WCA reduction from ∼116° (untreated) to ∼5° after 48 h in 5 M NaOH at 60 °C [50], while Bright et al. observed a decrease to ∼7.6° after 4 h in 1 M NaOH under hydrothermal conditions, compared to ∼76° for untreated samples [32]. Such enhanced wetting may support the infiltration and immobilization of antimicrobial agents within the nanostructured surface. It may also influence the initial interaction of bacteria, proteins, and cells with the material by improving liquid penetration into the surface structures. However, wettability cannot be considered independently of the accompanying changes in topography, oxide‐layer formation, and near‐surface chemistry. The observed biological response is therefore likely governed by the combined influence of these surface properties rather than by roughness or hydrophilicity alone.

#### 3.1.2 Elemental Composition

**FIGURE 4:**
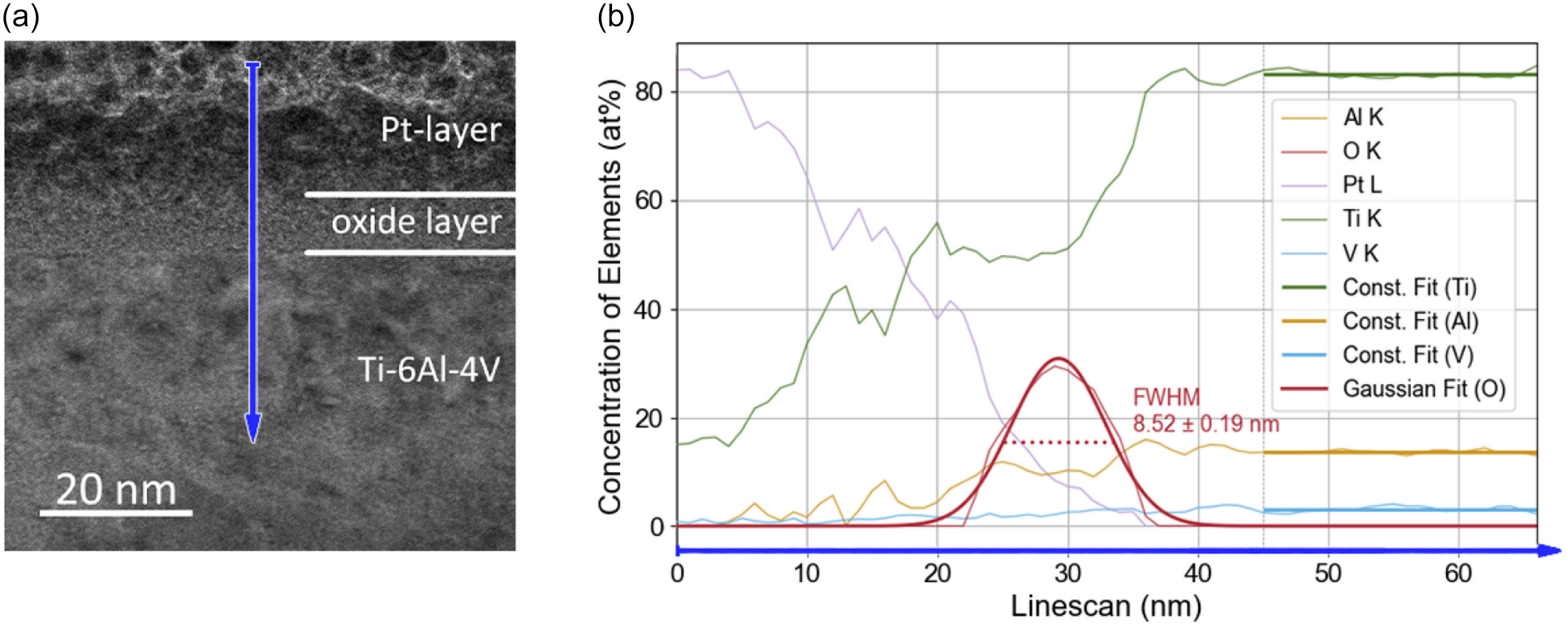
(a) TEM image of the cross‐sectional profile of an unetched Ti–6Al–4V lamella. The blue arrow indicates the trajectory of the TEM–EDX linescan shown in (b). (b) Elemental distributions of the most relevant elements are presented in atomic percent. A Gaussian fit was applied to the oxygen signal to estimate the thickness of the oxygen‐enriched surface layer.

To investigate the microstructure and elemental composition of the unetched material, a polished Ti–6Al–4V sample was prepared for TEM. Figure 4a displays a TEM image of the prepared lamella, acquired using a JEOL Neoarm 200F operated in bright‐field mode. The platinum layer visible in the upper portion of the image originates from the sample preparation process and is not intrinsic to the material. Directly beneath this layer, a structurally distinct band labeled as an oxide layer is present. Below this region, the bulk Ti–6Al–4V material is visible.

The spontaneous formation of a titanium dioxide (TiO_2_) layer is essential for the biocompatibility of titanium, as it provides chemical stability and renders the surface bioinert [51, 52]. Upon exposure to air or aqueous media, titanium surfaces form a native oxide layer within seconds, typically measuring 5–10 nm in thickness and predominantly composed of TiO_2_ [53, 55]. Since no subsequent heat treatment or annealing was performed, the etched structures are expected to be in an amorphous state [56].

This passivation layer effectively suppresses further oxidation, even under moderate thermal or humid conditions. If mechanically damaged, the underlying metallic titanium is immediately repassivated through interaction with oxygen or water [52]. This self‐healing capability is a key factor contributing to the material’s excellent corrosion resistance under physiological conditions.

To determine the thickness of the oxide layer, a TEM–EDX linescan was performed over a range of 65 nm. The scan trajectory is indicated by the blue arrow in Figure 4a. Figure 4b displays the resulting elemental distribution in atomic percent. A Gaussian fit was applied to the oxygen signal, and the full‐width‐at‐half‐maximum FWHM was used to estimate the oxide layer thickness, yielding a value of 8.5 ± 0.2 nm. This result is consistent with previously reported values [54]. No lateral elemental inhomogeneities were observed along the TEM–EDX linescan, suggesting a laterally uniform oxide layer composition within the analyzed region. The Pt signal reveals a gradual penetration of the element into the amorphous oxide layer, which is attributed to ion implantation during sample preparation. The bulk material is unaffected by Pt.

To determine the elemental composition of the bulk Ti–6Al–4V, constant fits were performed on Ti, Al, and V signals starting laterally through the TEM lamella, beginning approximately 45 nm below the original sample surface. Resulting in 88.4 ± 1.0 wt% of Ti, 8.2 ± 0.9 wt% of Al, and 3.5 ± 0.6 wt% of V. The higher Al concentration of Al at the cost of Ti and V indicates that the lamella might be cut out from an Al‐rich secondary phase of the material.

Upon normalizing the elemental signals of Ti, Al, and V within the oxide region, a minor enrichment of Al (9.5 ± 1.0 wt%) at the expense of Ti becomes evident. Both Ti and Al exhibit a strong tendency to oxidize. The slightly higher oxidation tendency and greater thermodynamic stability of Al_2_O_3_ compared to TiO_2_ can account for the incorporation of Al into the passivation layer, despite the overall higher availability of Ti for oxidation [57]. Consequently, the resulting passive film represents an amorphous Ti–Al mixed oxide layer. These findings are consistent with the descriptions reported by Leyens [58].

**FIGURE 5:**
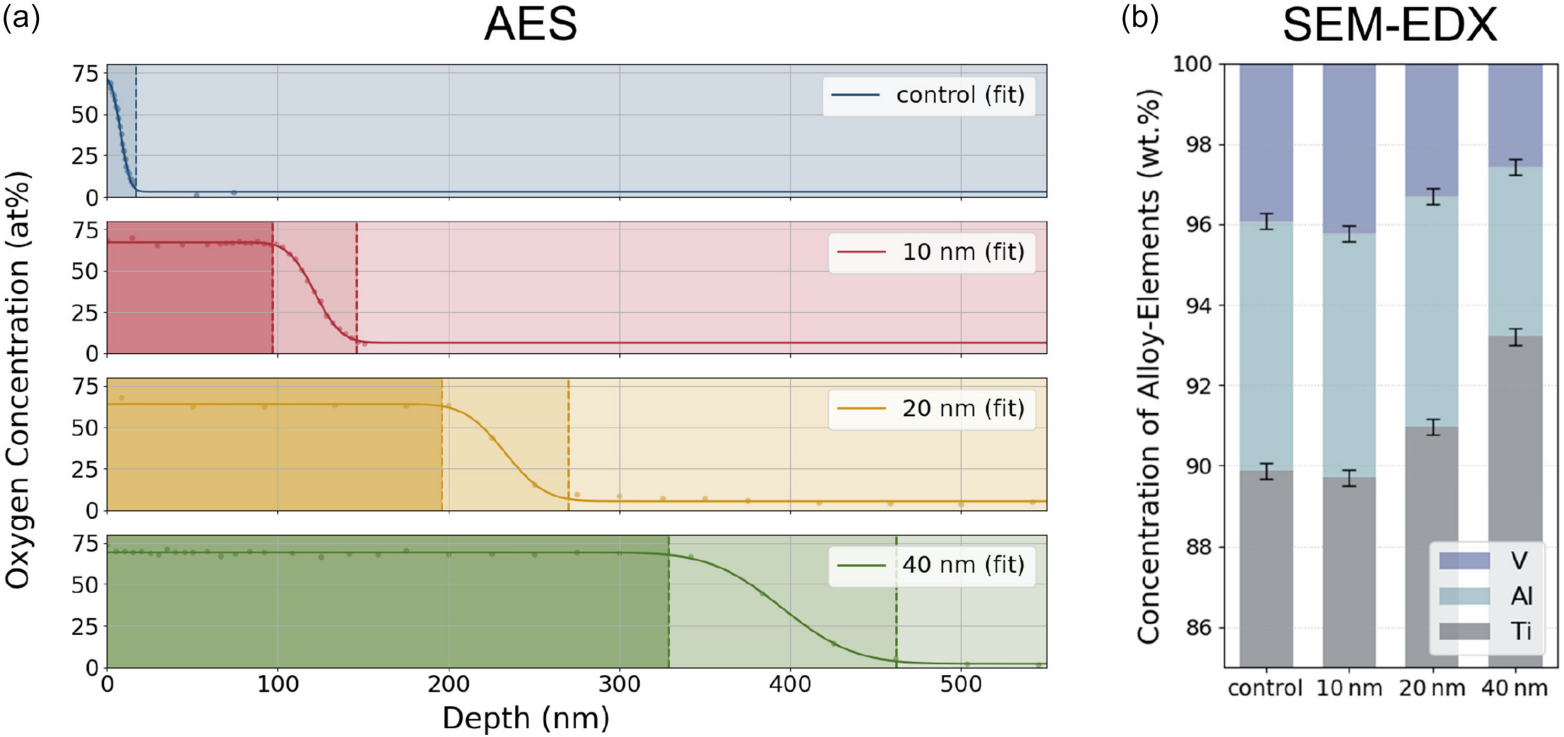
(a) Oxygen concentration profiles in atomic percent obtained by AES measurements for the four sample groups. The *x*‐axis is identical for all graphs. Estimated oxide and transition layer regions are indicated by shaded areas with different opacities. (b) Stacked bar chart showing the elemental composition (wt%) for all sample groups obtained by SEM–EDX measurements at 10 keV. The *y*‐axis is restricted to 85–100 wt% to highlight minor compositional changes.

Depth profiling by AES was employed to assess near‐surface oxygen concentration changes induced by etching (Figure 5a). The resulting values are summarized in Table 2. The AES depth profiles revealed a systematic increase in both oxide and transition layer thickness with increasing etching duration. The gradual transition in the oxygen signal suggests a diffuse oxide–metal interface, as observed in substoichiometric TiO_*x*_ transition regions [59]. Oxide thickness (*d*) and surface roughness (*R*_q_) increased concurrently, from *d* ≈ 0 nm and *R*_q_ = 0.24 nm in unetched controls to *d* = 329 nm and *R*_q_ = 36.7 nm after 24 h etching, indicating that oxidation and vertical structure growth proceed simultaneously. The control sample showed only a negligible native oxide layer with a measured thickness of 0.0 ± 0.4 nm, consistent with a passivated Ti surface, while the 40 nm group showed a substantially thicker oxide and transition zone. These findings on nanostructure thickness are consistent with the SEM cross‐sections obtained after FIB milling (see Figure 1e–h).

**TABLE 2:** Elemental composition of the four sample groups.

Sample group	AES		SEM–EDX (10 keV)		
	Oxide layer, nm	Transition layer, nm	Ti, wt%	Al, wt%	V, wt%
Control	0.0 ± 0.4	17.5 ± 0.8	89.9 ± 0.2	6.1 ± 0.2	3.9 ± 0.2
10 nm	96.8 ± 1.0	49.4 ± 1.9	89.7 ± 0.2	6.1 ± 0.2	4.2 ± 0.2
20 nm	195.9 ± 4.6	74.1 ± 8.6	91.0 ± 0.2	5.7 ± 0.2	3.3 ± 0.2
40 nm	328.6 ± 4.5	133.2 ± 8.3	93.2 ± 0.2	4.2 ± 0.2	2.6 ± 0.2

*Note:* The left side reports the thicknesses of the oxide and transition layers, respectively, as determined from AES spectra. The three values on the right present the mass percentages of the detected elements based on SEM–EDX measurements over an area of 1050 µm^2^ at an excitation energy of 10 keV. While AES depth profiles provide vertical oxide gradients, SEM–EDX reflects the laterally averaged elemental distribution.

To exclude the possibility of residual sodium contributing to antimicrobial behavior, AES was also used to quantify surface sodium concentrations. For all sample groups, the Na content remained below 0.1 at%, confirming that any biological effects observed in subsequent experiments are attributable solely to topographical modifications, not chemical residue.

To investigate potential changes in the bulk composition of Ti–6Al–4V induced by progressive etching, SEM–EDX was performed on all four sample groups. To selectively probe the near‐surface oxide region, low excitation energies are generally preferred to minimize electron beam penetration depth. Area scans covering 1050 µm^2^ were acquired at acceleration voltages of 5, 10, and 15 kV to assess both lateral homogeneity and statistical variance. At 5 kV, the excitation energy is only slightly above the K‐shell ionization threshold of vanadium (∼4.95 keV), which results in low X‐ray yield. In addition, low‐energy X‐rays such as Al K*α* are strongly attenuated in the sample and detector window. As a consequence, no vanadium signal was detected, and aluminum was substantially underestimated (2.9 wt%). This condition was therefore excluded from further analysis.

At 10 keV, the excitation conditions were found to provide a suitable compromise between penetration depth and sufficient excitation of all alloy constituents. According to the Kanaya–Okayama equation and corresponding Monte Carlo simulations, the average electron penetration depth was estimated to be approximately 250 nm [60, 61]. The results are also visualized in Figure 5b, which reveals a gradual relative increase in titanium and a corresponding decrease in aluminum and vanadium with etch depth, supporting our hypothesis that surface‐selective dissolution occurs during alkaline treatment. For alkaline treated samples, the distribution at 15 keV more closely resembles that of a control sample, which is attributed to the larger penetration depth, as the counts here are increasingly originating from underneath the oxide layer [60]. A conservative uncertainty estimate of ±0.2 wt% was adopted based on instrumental specifications and literature values for standardless quantification.

As shown in Table 2, the elemental composition of the control samples closely matches the expected values for Ti–6Al–4V, as reported by the manufacturer. With increasing etching time, a systematic reduction in the detected Al and V content is observed, while the Ti concentration correspondingly increases. This trend, recorded at constant acceleration voltage (10 keV), suggests a preferential dissolution of Al and V during alkaline treatment, indicating that these alloying elements are more susceptible to etching than the Ti matrix. This may be attributed to selective dissolution processes during alkaline treatment: Aluminum stabilizes the *α*‐(Ti–Al) phase, while vanadium stabilizes the *β*‐(Ti–V) phase. It is reported that vanadium‐rich *β*‐regions show higher chemical reactivity and are preferentially attacked under alkaline conditions, leading to local depletion of V and Al [62]. Furthermore, the solubility of ${\mathrm{Al}(\mathrm{OH})}_{4}^{-}$ and ${\mathrm{VO}}_{4}^{3-}$ species in NaOH solutions is significantly higher than that of titanium oxides, promoting leaching of these elements into the etchant [63]. These findings support the interpretation that alkaline etching induces a subtle but systematic compositional shift in favor of titanium, reflecting both structural and chemical selectivity of the process.

### 3.2 Ti–6Al–4V Surface Infection

#### 3.2.1 *S. aureus* USA300 WT (Gram Positive)

To assess bacterial adherence of *S. aureus* USA300 wild type (WT), a defined bacterial inoculum was incubated with Ti–6Al–4V surfaces showing controlled nanoroughness levels of 0.2 nm (unetched control), 10, 20, and 40 nm for 1 h or 24 h, as described in Section 2. Adherence was quantified by measuring the surface area covered (µm^2^) in microscopy images.

**FIGURE 6:**
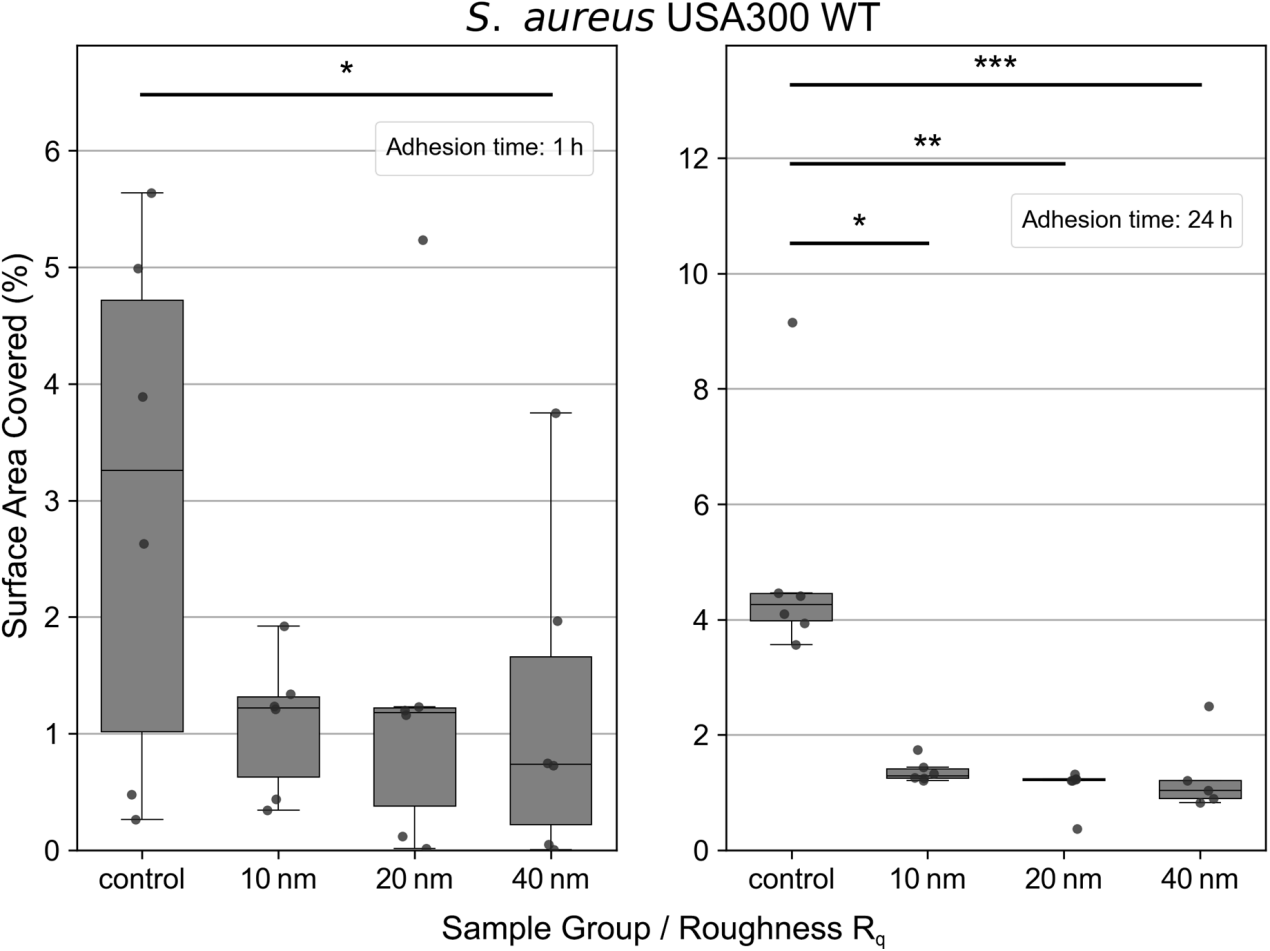
Boxplots showing surface area covered (%) of *S. aureus* USA300 WT on etched Ti–6Al–4V surfaces with nominal roughness values of 0.2 nm (control), 10, 20, and 40 nm after adhesion times of 1 h (left) and 24 h (right). Boxplots display medians and interquartile ranges; whiskers represent 1.5 × IQR; individual points correspond to per‐sample values. Statistically significant differences between roughness groups were determined using the Kruskal–Wallis test (*α* = 0.05) followed by Dunn’s post hoc test with Holm–Šidák correction. Significance levels: *p* < 0.05 (*), *p* < 0.01 (**), *p* < 0.001 (***).

After 1 h, *S. aureus* showed a statistically significant reduction in adhesion on all etched surfaces compared to the unetched control, indicating an early influence of surface topography on initial colonization (Figure 6, left panel). At 24 h, this difference became more pronounced. All etched surfaces displayed statistically significant lower *S. aureus* coverage than unetched control surfaces with *p* < 0.05 (Figure 6, right panel). The effect was consistent across all replicates, confirming the substantial impact of surface nanoroughness in reducing bacterial colonization, even after 24 h exposure. Representative fluorescence images corresponding to the quantitative adhesion analyses for this and the following bacterial strains are provided in the Supporting Information (Figure S1).

#### 3.2.2 *S. aureus* USA300 WT GFP (Gram Positive)

**FIGURE 7:**
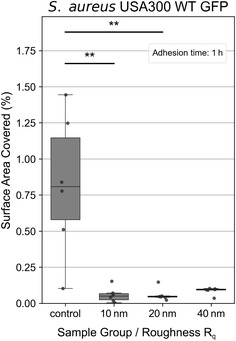
Boxplots showing surface area covered (%) of *S. aureus* USA300 WT GFP on etched Ti–6Al–4V surfaces with nominal roughness values of 0.2 nm (control), 10, 20, and 40 nm after an adhesion time of 1 h. Boxplots display medians and interquartile ranges; whiskers represent 1.5 × IQR; individual points correspond to per‐sample values. Statistically significant differences between roughness groups were determined using the Kruskal–Wallis test (*α* = 0.05) followed by Dunn’s post hoc test with Holm–Šidák correction. Significance level: *p* < 0.01 (**).

This GFP‐expressing strain differs from USA300 WT only by carrying a constitutively expressed green fluorescent protein marker, while the wild‐type strain has no fluorescent reporter. Using this viability‐based fluorescence imaging method, we observed a pronounced and statistically significant reduction in *S. aureus* USA300‐GFP adhesion on nanorough surfaces compared to the unetched control. The unetched control showed the highest surface colonization. Increasing nanoroughness from 10 to 40 nm after 1 h reduced bacterial attachment (Figure 7). Additional 24 h data from the GFP experiments are shown in Figure S2. These findings reinforce the inhibitory effect of nanoroughness on the early adhesion of viable *S. aureus*. They also validate our previous observations using an independent and biologically direct quantification approach.

#### 3.2.3 *S. epidermidis* ATCC12228 (Gram Positive)

**FIGURE 8:**
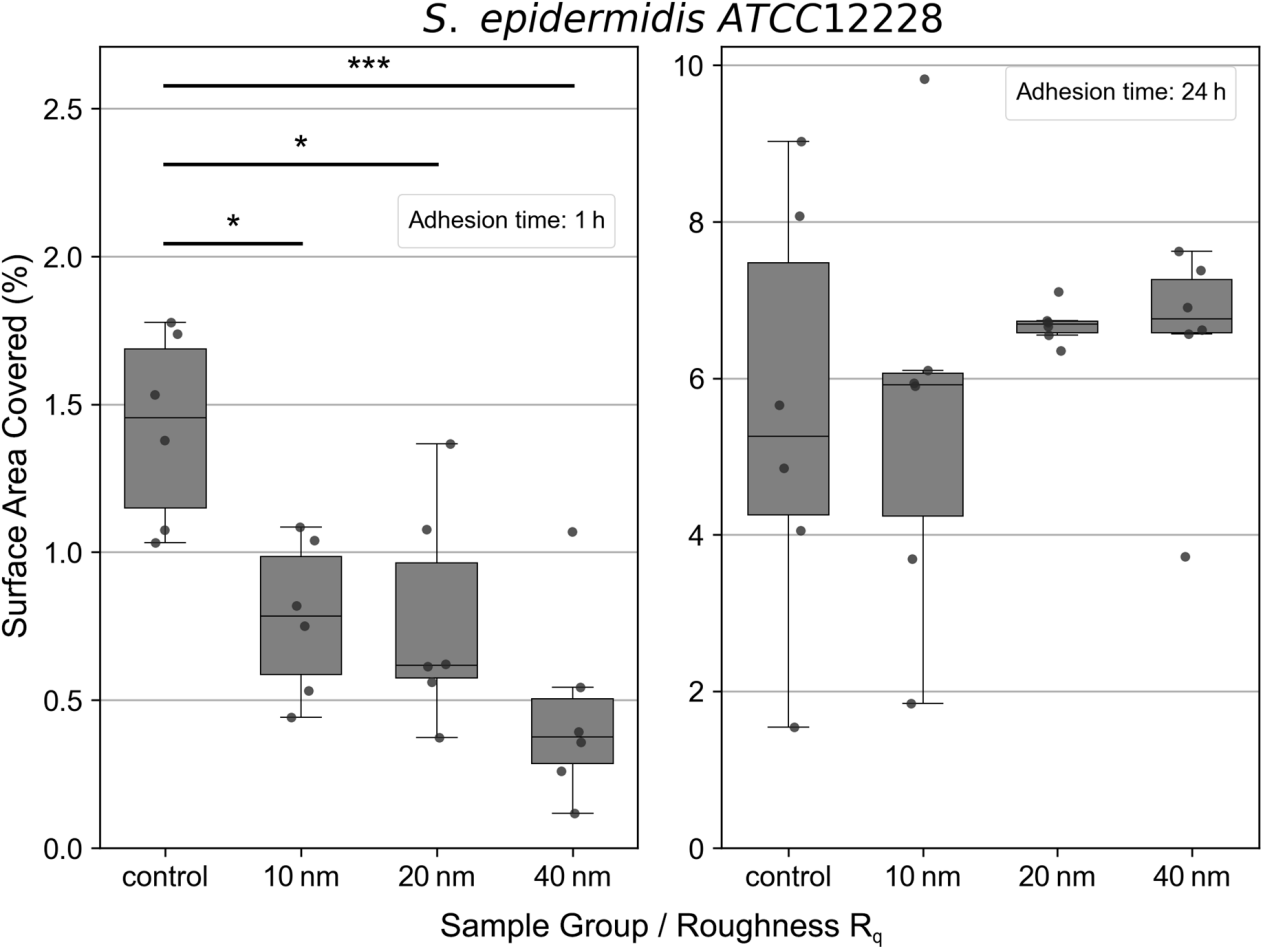
Boxplots showing surface area covered (%) of *S. epidermidis* ATCC12228 on etched Ti–6Al–4V surfaces with nominal roughness values of 0.2 nm (control), 10, 20, and 40 nm after adhesion times of 1 h (left) and 24 h (right). Boxplots display medians and interquartile ranges; whiskers represent 1.5 × IQR; individual points correspond to per‐sample values. Statistically significant differences between roughness groups were determined using the Kruskal–Wallis test (*α* = 0.05) followed by Dunn’s post hoc test with Holm–Šidák correction. Significance levels: *p* < 0.05 (*), *p* < 0.001 (***).

To assess bacterial adhesion, another clinically relevant Gram‐positive species, *S. epidermidis* ATCC12228, was used alongside *S. aureus*. Bacteria were incubated on Ti–6Al–4V surfaces with defined nanoroughness, like in previous experiments, and analyzed after 1 and 24 h. Quantitative image analysis after 1 h of incubation revealed a marked reduction in *S. epidermidis* adhesion on nanorough surfaces (10–40 nm) compared to the unetched control (Figure 8, left panel). This effect was most pronounced at 20 and 40 nm, suggesting that nanoscale surface features can significantly impair initial bacterial attachment. However, after 24 h of incubation, this difference was no longer evident (Figure 8, right panel). Bacterial coverage increased over time and appeared similar across all surface conditions, indicating that *S. epidermidis* can adapt to surface topographies and eventually colonize even less favorable surfaces.

#### 3.2.4 *E. coli* 10724 (Gram Negative)

**FIGURE 9:**
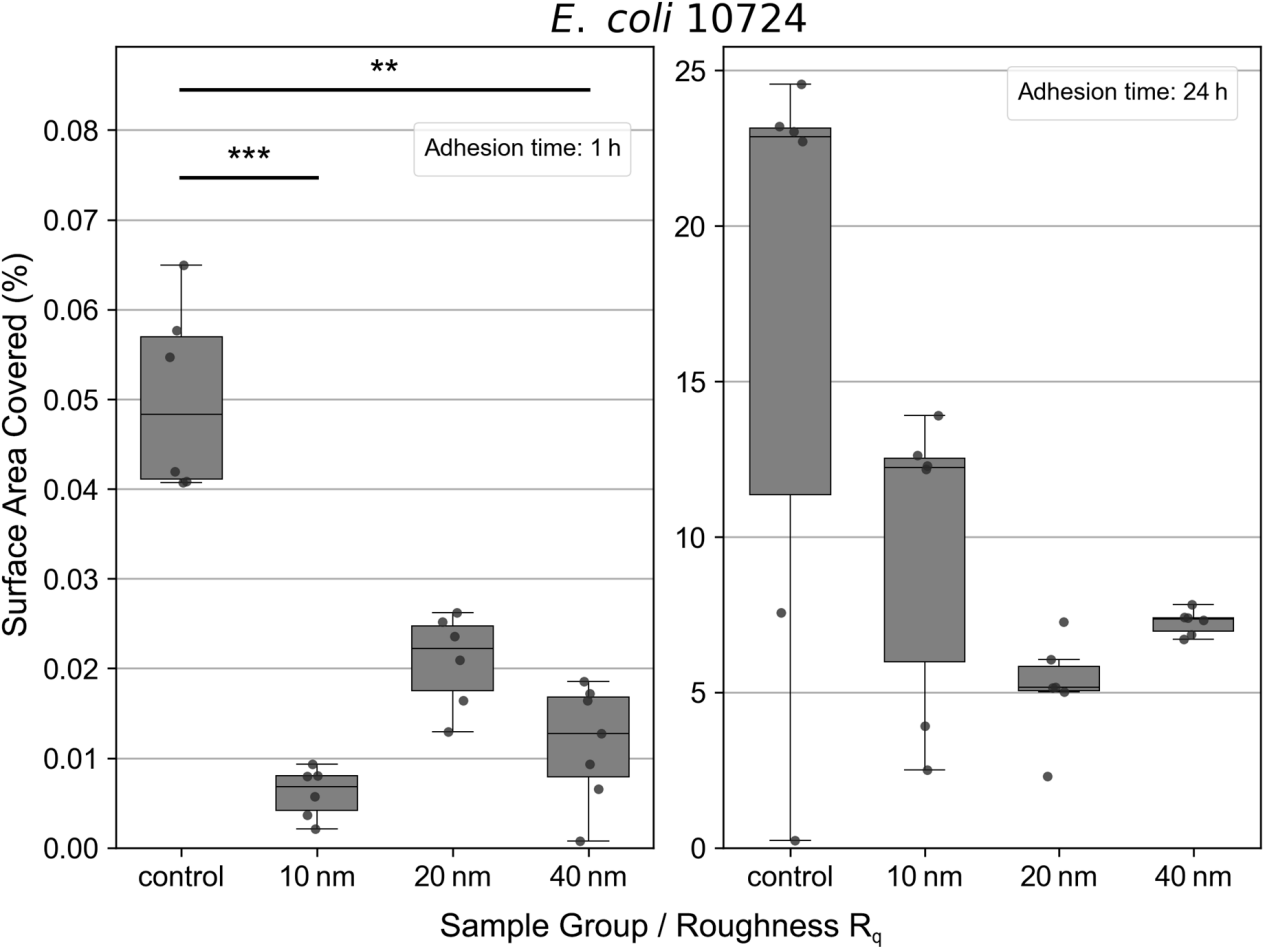
Boxplots showing surface area covered (%) of *E. coli* 10724 on etched Ti–6Al–4V surfaces with nominal roughness values of 0.2 nm (control), 10, 20, and 40 nm after adhesion times of 1 h (left) and 24 h (right). Boxplots display medians and interquartile ranges; whiskers represent 1.5 × IQR; individual points correspond to per‐sample values. Statistically significant differences between roughness groups were determined using the Kruskal–Wallis test (*α* = 0.05) followed by Dunn’s post hoc test with Holm–Šidák correction. Significance levels: *p* < 0.01 (**), *p* < 0.001 (***).

Including both Gram‐positive and Gram‐negative bacteria is important in BAI research, since Gram‐negative species differ from Gram‐positive bacteria in cell wall structure and adhesion mechanisms, and they account for a substantial proportion of BAIs. To address this, we included *E. coli* 10724 in our experiments. This clinically isolated strain increases the clinical relevance of the study. As described previously, the bacteria were incubated with Ti–6Al–4V samples exhibiting different levels of nanoroughness. After 1 h of incubation, *E. coli* adherence was significantly reduced on the rougher surfaces (Figure 9, left panel). This indicates that increased surface roughness can impair adhesion in Gram‐negative species despite their distinct attachment mechanisms. These findings align with our earlier results in *S. aureus* and *S. epidermidis*. After 24 h, only a nonsignificant trend toward reduced adhesion was observed (Figure 9, right panel).

#### 3.2.5 SEM Analysis of Bacterial Adhesion at 1 and 24 h Incubation Times

**FIGURE 10:**
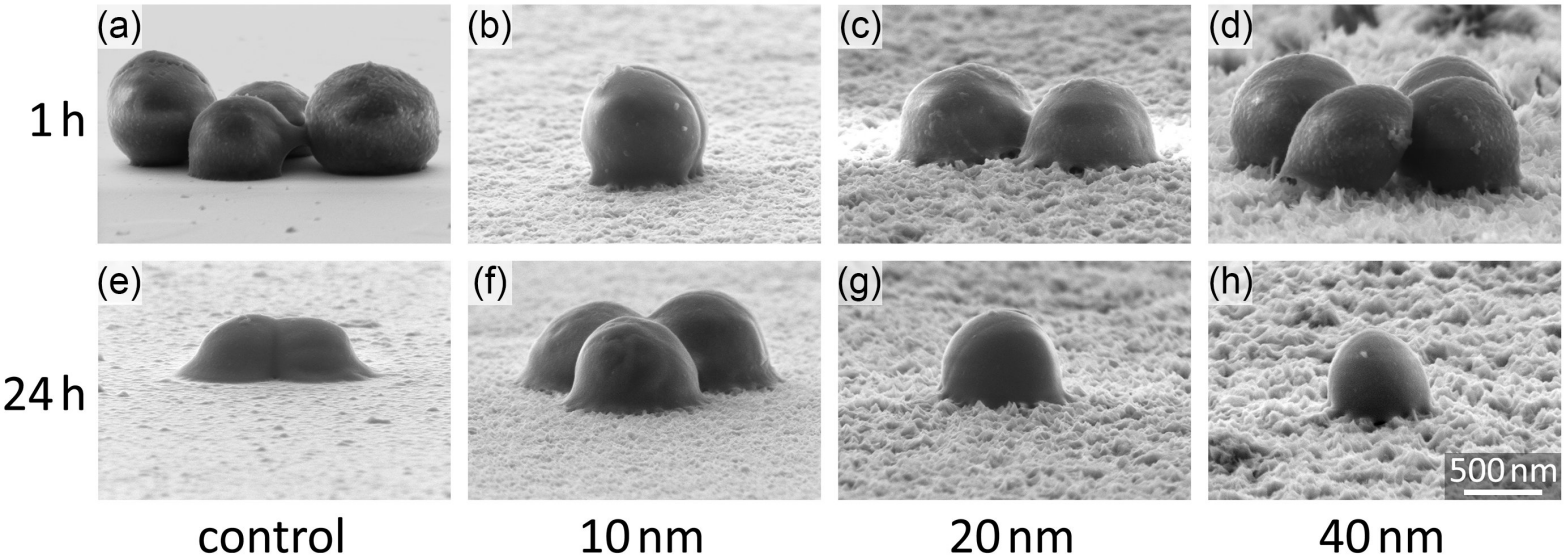
SEM images of *S. aureus* USA300 WT adhesion on Ti–6Al–4V surfaces with increasing nanoroughness after 1 h (a–d) and 24 h (e–h) of incubation. The unetched control (a,e) shows well‐spread cells with broad surface contact, indicating strong attachment. With increasing roughness (b–d,f–h), cells appear less spread. The scale bar in (h) applies for all the images of this figure.

To complement the quantitative analysis of bacterial adherence, SEM was performed on representative samples to visualize *S. aureus* interactions with Ti–6Al–4V surfaces of varying nanoroughness. Figure 10a–d presents SEM images taken after 1 h of incubation, while images (e–h) show the corresponding surfaces after 24 h. Across both time points, the SEM images primarily showed individually adhered bacterial cells rather than large aggregates, enabling the assessment of single‐cell attachment characteristics. Tilted‐view SEM imaging provides an additional perspective that enhances the qualitative assessment of bacterial–surface interactions in this study.

In the 1‐h images (a–d), the unetched control (a) showed bacteria with broad and uninterrupted surface contact, indicating a large interfacial area available for adhesion. As surface roughness increased (b–d), bacterial cells appeared less well‐spread, often interacting with the surface at only a few discrete points. In some cases, cells adopted a tilted (d) or partially suspended orientation, likely due to limited surface contact or entrapment within surface features, in contrast to the uniform attachment seen on the unetched control.

The 24‐h SEM images (e–h) showed adherence patterns similar to those at 1 h, with etched surfaces continuing to display fewer firmly attached bacteria and a more limited cell–surface interface. In image (e) 24 h control, individual cells appear more aggregated than in 1 h. They form a more flattened skirt‐like structure and an apparent greater contact area with the surface.

**FIGURE 11:**
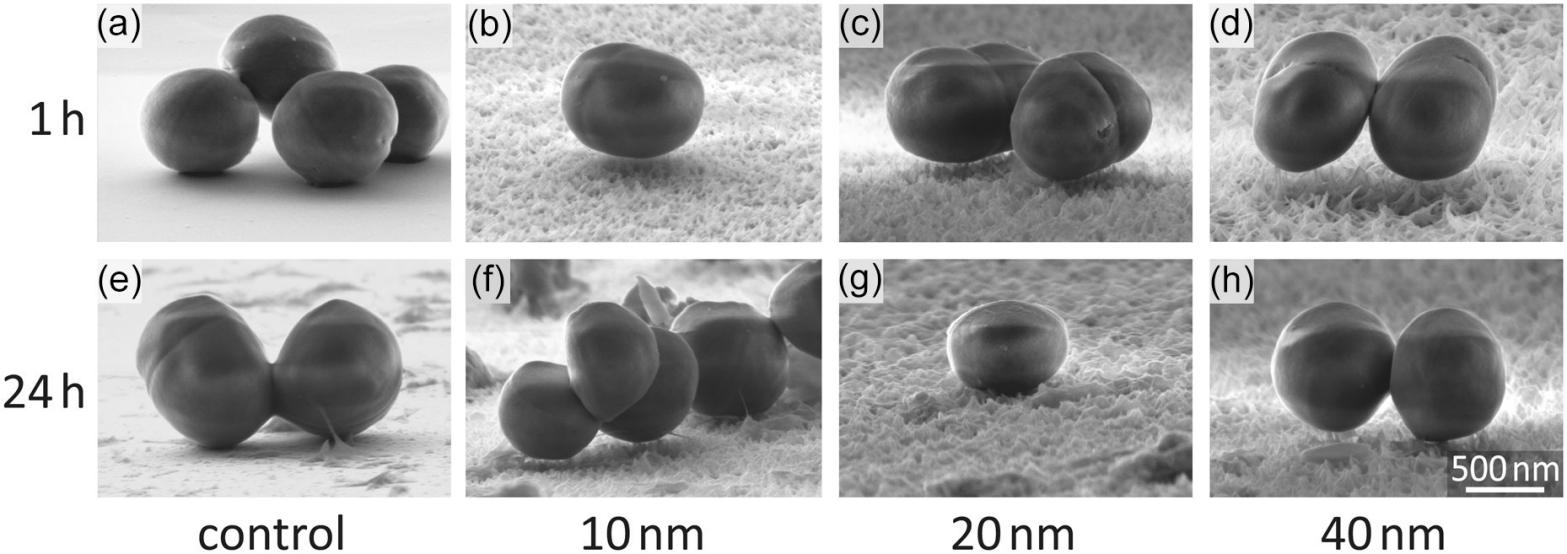
SEM images of *S. epidermidis* ATCC12228 adhesion on Ti–6Al–4V surfaces with increasing nanoroughness after 1 h (a–d) and 24 h (e–h) of incubation. The unetched control (a,e) shows broad surface contact, leading to a stronger attachment. With increasing roughness (b–d,f–h), there is a visible gap between the bacteria and the surface. The scale bar in (h) applies for all the images of this figure.

We then examined *S. epidermidis* adhesion to the surfaces at the single‐cell level, following the same approach used for *S. aureus*, shown in (Figure 11). When comparing *S. epidermidis* with *S. aureus*, we did not observe the same well spread structure on the 24 h control sample Figures 10e and 11e. However, we still observed decreased intrastrain bacterial contact area after 1 and 24 h with increasing surface roughness, consistent with the observations for *S. aureus*. These findings suggest that the modified topographies reduce effective bacterial contact area.

**FIGURE 12:**
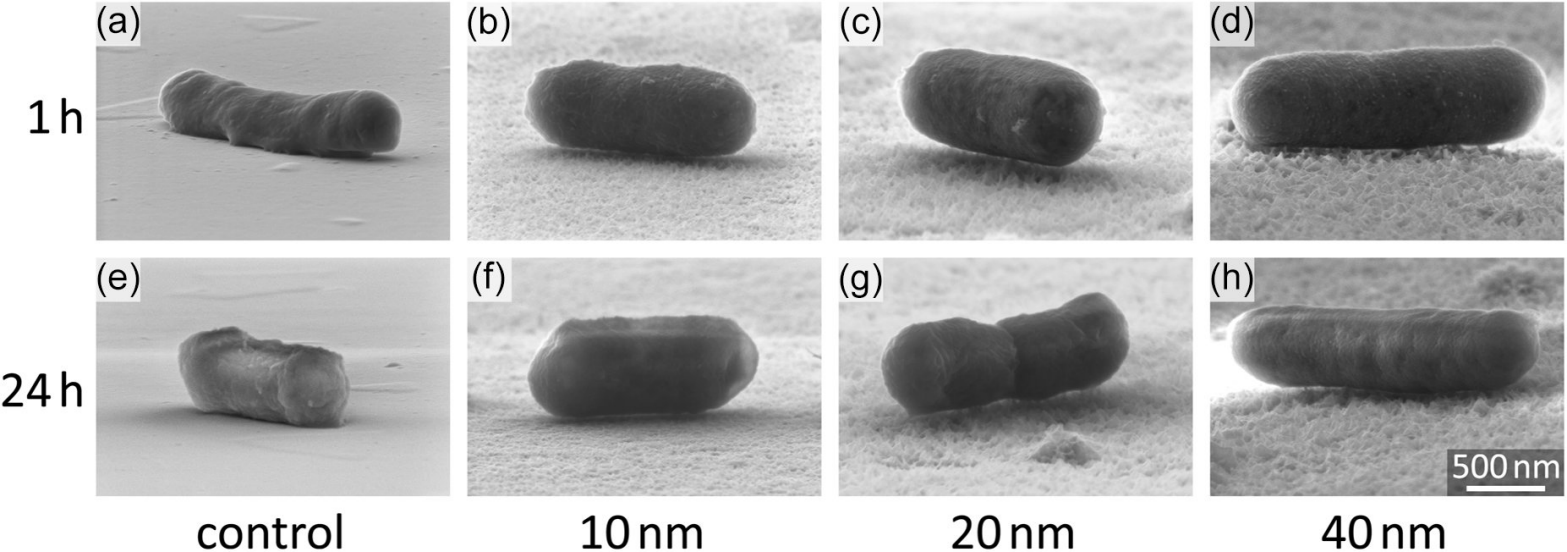
SEM images of *E. coli* adhesion on Ti–6Al–4V surfaces with increasing nanoroughness after 1 h (a–d) and 24 h (e–h) of incubation. The unetched control (a,e) shows broad surface contact, leading to a stronger attachment. With increasing roughness (b–d,f–h), there is a visible gap between the bacteria and the surface. The scale bar in (h) applies for all the images of this figure.

Like the analysis performed for *S. aureus* and *S. epidermidis*, SEM was used to assess and visualize the adhesion behavior of *E. coli*. Figure 12a–d shows SEM images taken after 1 h of incubation, illustrating the bacterium’s interaction with surfaces of increasing roughness. On the unetched control (a), *E. coli* displayed broad and continuous contact with the samples, indicating a well‐established interface. In contrast, images of rougher surfaces revealed a distinct separation or gap between the bacterial cell and the surface, suggesting reduced physical contact or partial suspension.

**FIGURE 13:**
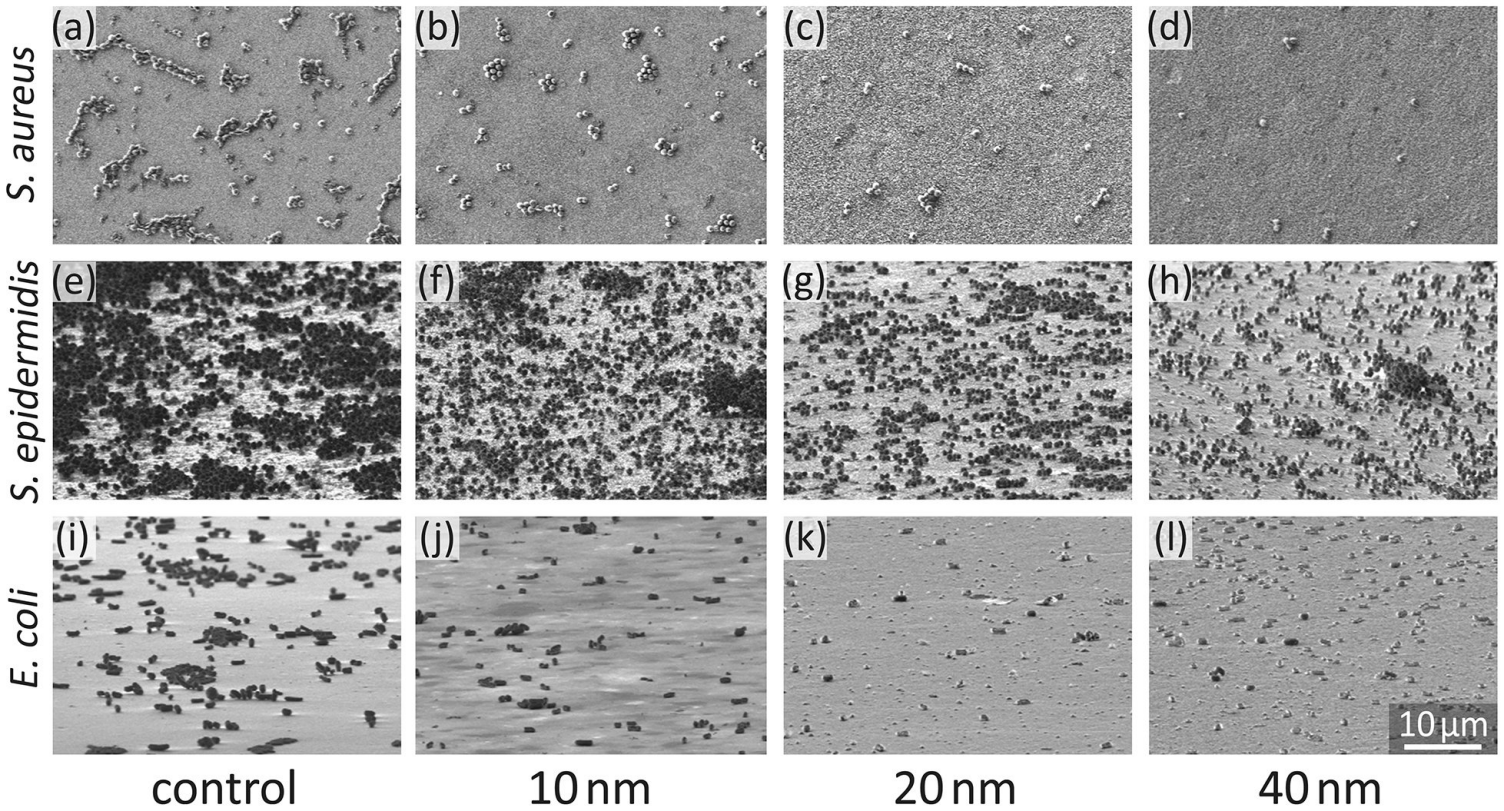
SEM images of *S. aureus* USA300 WT (a–d), *S. epidermidis* ATCC12228 (e–h) and *E.coli* 10724 (i–l) adhesion on Ti–6Al–4V surfaces with increasing nanoroughness after 24 h incubation. Vertical stacking of S. epidermidis (e–h) and the development of a multilayered organization of bacterial aggregates architecture is highlighted by tilted views. The scale bar in (l) applies for all the images of this figure.

SEM observations indicated that the modified surfaces reduced the effective contact area of *S. epidermidis* after 24 h. However, this qualitative observation was not consistent with the previously obtained quantitative results, which showed no statistically significant differences in *S. epidermidis* adhesion on modified surfaces at 24 h. Therefore, we further compared *S. aureus* and *S. epidermidis* not only at the cell–surface interface but also in terms of cell–cell interactions to assess potential differences in their spatial organization on the surfaces. Specifically, we examined whether *S. epidermidis* exhibits vertical organization of bacterial aggregates via SEM imaging. Figure 13 presents a comparison of *S. aureus* (a–d) and *S. epidermidis* (e–h) after 24 h. The tilted‐view images of *S. epidermidis* (e–h) reveal that this species not only colonizes the surface effectively but also forms multilayered aggregates, indicating an increased capacity for cell recruitment and vertical accumulation of surface‐associated bacterial aggregates. In contrast, *S. aureus* developed comparatively flatter microcolonies with limited vertical structure.

A similar pattern was observed in the 24 h images (e–h). Unlike *S. aureus*, *E. coli* did not show a pronounced or well‐spread morphological change under control conditions after 24 h. This reflects the fundamental differences in adhesion strategies between the two strains.

#### 3.2.6 Bacterial Responses to Nanostructured Surfaces

Nanoscale surface modifications on Ti–6Al–4V influenced the early adhesion of all tested bacterial strains. Bright et al. demonstrated that nanospike‐like Ti–6Al–4V surfaces suppress long‐term biofilm formation mainly through mechanical rupture of attached bacteria [44]. Our findings complement these observations by showing that nanostructured Ti–6Al–4V surfaces already impair bacterial attachment during the initial adhesion phase. Similar antibacterial trends were reported by Valdez‐Salas et al. for TiO_2_ nanotube arrays, although their ordered fluoride‐containing anodized surfaces differ substantially from the mesh‐like nanoporosity investigated here [41]. Together, these studies highlight the importance of nanoscale topography in modulating bacterial–surface interactions.

Both *S. aureus* and *S. epidermidis* showed significantly reduced adhesion on nanorough surfaces after 1 h, while *E. coli* exhibited a similar trend, indicating that early bacterial adhesion is highly sensitive to nanoscale surface architecture.

After 24 h, however, clear species‐specific differences became apparent. *S. epidermidis* maintained relatively high surface coverage across all conditions, whereas *S. aureus* adhesion remained significantly reduced on nanorough surfaces. These differences likely reflect distinct adhesion and virulence strategies. *S. epidermidis* relies mainly on surface‐associated adhesins such as AtlE and Aap and commonly produces the polysaccharide intercellular adhesin PIA/PNAG, which promotes strong cell–cell interactions and multilayered bacterial aggregates [43, 64]. In contrast, *S. aureus* expresses a broader range of virulence‐associated adhesins, including MSCRAMMs, protein A, fibronectin‐binding proteins, and extracellular DNA, while PIA/PNAG contributes less consistently to biofilm formation [43, 65].

SEM analysis further showed that *S. epidermidis* developed pronounced multilayered aggregates after 24 h, whereas *S. aureus* formed comparatively flatter microcolonies (Figure 13). Since *S. epidermidis* pathogenicity is strongly linked to biofilm formation, this early vertical organization may facilitate rapid persistence on biomaterial surfaces. In contrast, *S. aureus* possesses additional virulence strategies beyond biofilm formation, which may explain its delayed development of multilayered aggregates under the investigated conditions [66]. These findings further emphasize the importance of including multiple clinically relevant bacterial species in biomaterial studies, as surface modifications may affect individual pathogens differently even within the same bacterial family.

The inclusion of *E. coli* further demonstrated that nanoscale topography also influences Gram‐negative bacteria. While adhesion was significantly reduced after 1 h, only a nonsignificant trend remained after 24 h. This may reflect the different adhesion strategy of rod‐shaped bacteria, which interact with surfaces primarily via pili or fimbriae rather than broad cell‐wall contact.

AES and SEM–EDX analyses confirmed that the observed biological effects were not attributable to residual chemical contaminants. Sodium concentrations remained below 0.1 at% in all groups, while oxide thickness increased progressively with etching. Together with the identical handling of all samples, these findings support a predominantly topography‐driven mechanism underlying the reduced bacterial adhesion observed on nanostructured Ti–6Al–4V surfaces.

### 3.3 SaOs‐2 Cells Exhibit Viable Adhesion and Morphological Adaptation

To evaluate initial osteoblast adhesion, SaOs‐2 cells were seeded on Ti–6Al–4V surfaces with increasing nanoroughness values like in previous experiments, as well as on glass slides serving as controls for cytotoxicity assay. After 24 h, the culture supernatant was collected for LDH assay to assess cytotoxicity. The cells on the substrates were fixed and stained with phalloidin (actin cytoskeleton) and DAPI (nuclei) as explained in Section 2.6.

**FIGURE 14:**
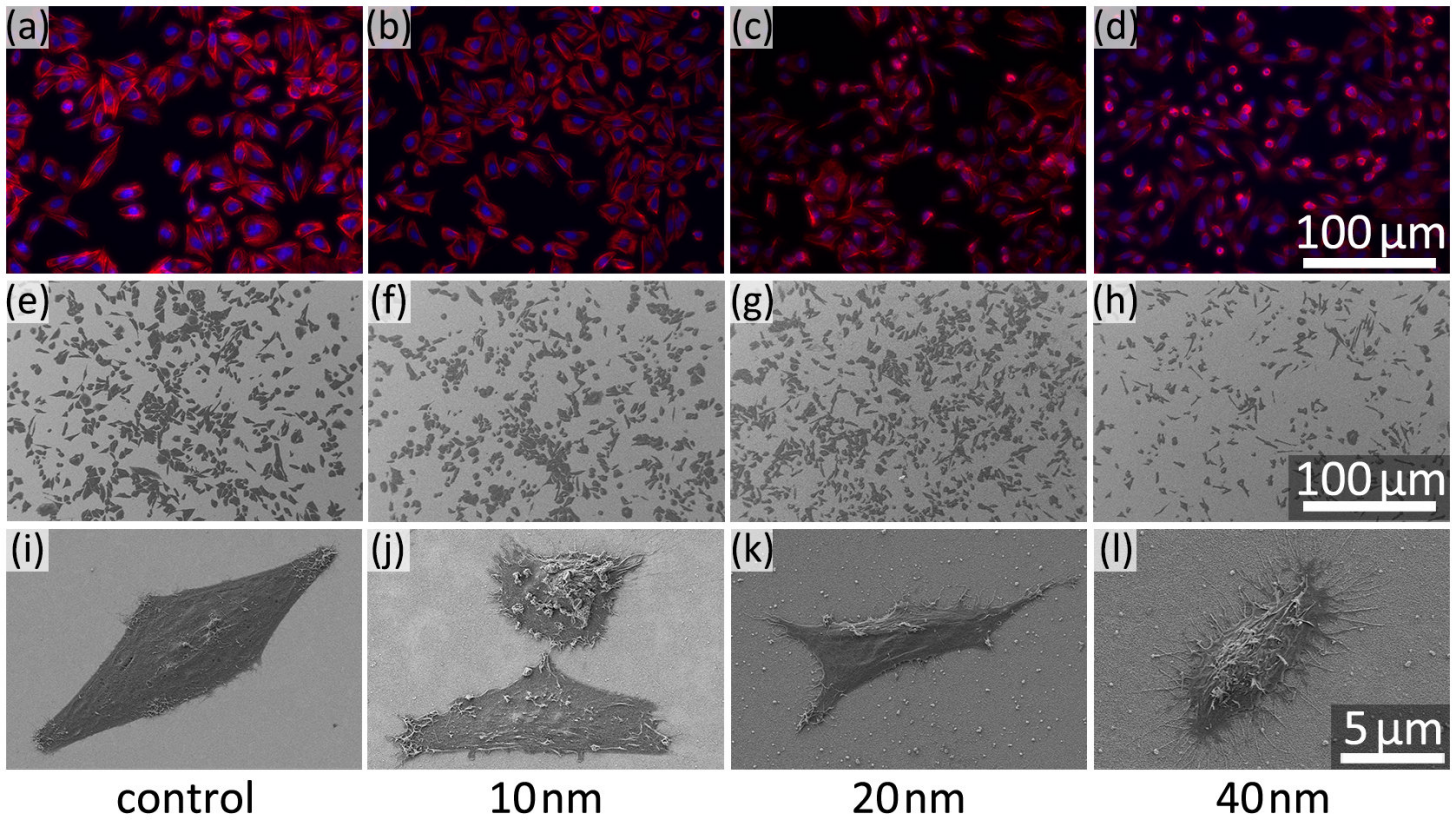
Adhesion and morphology of SaOs‐2 osteoblast‐like cells on Ti–6Al–4V surfaces with increasing nanoroughness. (a–d) Immunofluorescence images of phalloidin (actin) and DAPI (nuclei) staining show cell adhesion on different substrates. (e–h) SEM overviews illustrate overall cell coverage, and (i–l) higher magnification SEM images highlight single‐cell morphology. On 40 nm surfaces, cells appeared more spread with pronounced filopodial extensions, indicating stronger substrate interactions. The scale bars in (d), (h) and (l) apply for the images in the respective rows.

**FIGURE 15:**
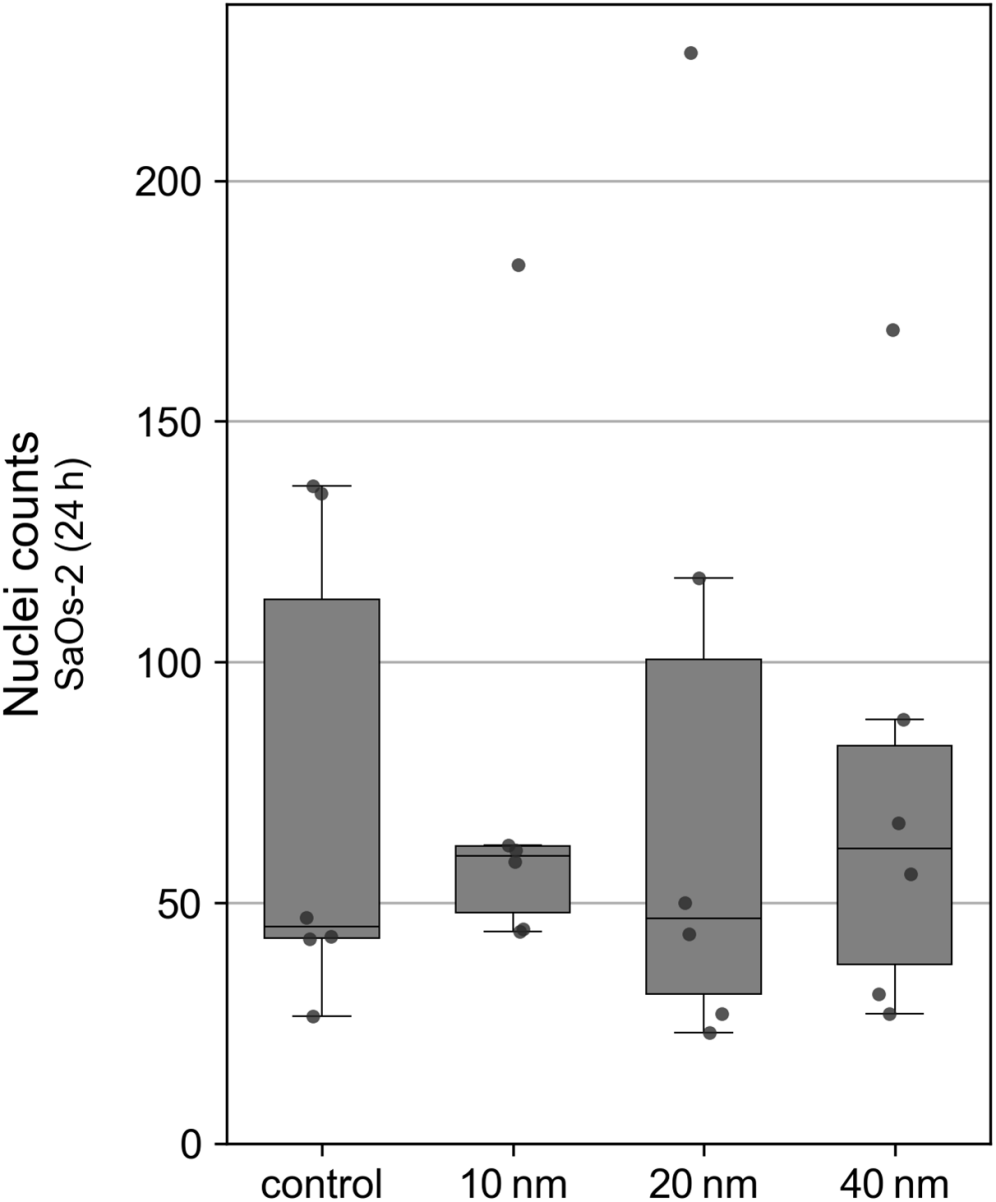
Quantification of cell coverage on etched Ti–6Al–4V surfaces with nominal roughness values of 0.2 nm (control), 10, 20, and 40 nm after adhesion time of 24 h based on DAPI‐labeled nuclei. Boxplots display medians and interquartile ranges; whiskers represent 1.5 × IQR; individual points correspond to per‐sample values. The data showed no statistically significant difference across the different roughness conditions.

Figure 14a–d shows representative immunofluorescence images of DAPI and phalloidin stained SaOs‐2 cells. Quantitative analysis of cell attachment was performed by measuring the nuclei counts of DAPI‐stained nuclei across 20 images per condition (Figure 15). We observed that the nuclei numbers were comparable across all Ti–6Al–4V surfaces. These findings indicate that SaOs‐2 cells adhered and proliferated similarly on both unetched and nanorough substrates, with no statistically significant differences observed between the tested conditions.

To qualitatively assess cell morphology and attachment, an additional set of samples under identical experimental conditions was prepared for SEM. Figure 14e–l presents a panel of representative SEM images.

Across all conditions, cells showed good attachment. On rougher Ti–6Al–4V surfaces, cells appeared more spread and displayed pronounced filopodial extensions Figure 14l. These morphological features suggest enhanced physical interaction with the substrate, despite the absence of statistically significant differences in cell number.

**FIGURE 16:**
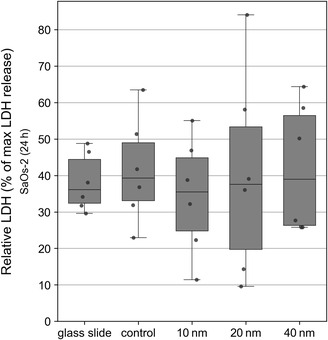
Cell viability was further confirmed by LDH cytotoxicity assay using culture supernatants collected after 24 h. No statistically significant cytotoxicity was detected for any Ti–6Al–4V surface or the glass slide control when compared to the maximum LDH release. Boxplots display medians and interquartile ranges; whiskers represent 1.5 × IQR; individual points correspond to per‐sample values.

To evaluate the cytocompatibility of the modified Ti–6Al–4V samples, LDH release in the culture supernatants was quantified after 24 h (Figure 16). Cells were cultured on surfaces with different nanoscale roughnesses, and an additional control (glass slides). LDH levels in the corresponding supernatants were then compared to the maximum LDH release provided by the assay’s positive control (lysed cells), allowing assessment of potential cytotoxic effects induced by the different surface conditions. No increase in LDH release was observed for any Ti–6Al–4V surface, demonstrating that none of the conditions induced cytotoxicity. These results support that all tested Ti–6Al–4V surfaces are biocompatible and equally supportive of osteoblastic cell growth.

In summary, the adhesion of osteoblast‐like cells was assessed after 24 h to capture an early integration stage, when cells have had sufficient time to establish stable focal adhesions. Quantitative analysis showed no statistically significant differences in the number of adhered cells between unetched control and rough surfaces. However, SEM imaging revealed distinct morphological differences. Cells on nanorough surfaces showed more extensive filopodia, this feature was not observed on the unetched control.

These findings indicate that, while overall cell numbers were comparable, nanorough surfaces facilitated more active physical interaction with the substrate. The differing responses of mammalian and bacterial cells likely reflect their biological characteristics. Bacteria can attach to biomaterial surfaces within minutes, driven by passive physicochemical interactions. Osteoblasts, in contrast, require more time to reorganize their cytoskeleton and form submicron focal adhesions that adapt to nanoscale features. As a result, bacterial adhesion is more immediately sensitive to nanoscale topography, whereas cellular responses become apparent at later stages.

This dual effect is critical in implant applications. Stable early osseointegration and infection prevention are both essential for long‐term success. Our results suggest that this balance is achievable: Nanorough surfaces significantly reduced early bacterial colonization while simultaneously supporting osteoblast adhesion and favorable cell morphology.

### 3.4 Nanoscale Contact Mechanics of Bacterial and Osteoblast Adhesion

Based on the quantitative adhesion data for bacteria and osteoblasts, together with the SEM observations shown in Figures 10, 11, 12, and 14, we derived a simple model to explain the observed behavior. To support this model quantitatively, we first assessed how the bacterial covered area related to the individual material and topographical parameters listed in Table 1. For this purpose, ordinary least squares (OLS) regression analyses were performed separately for each bacterial strain and surface parameter. This screening approach was used to identify whether classical roughness descriptors such as *R*_a_, *R*_q_, and *R*_sk_, or parameters describing the spatial distribution of nanoscale features, such as PtP and peak density, showed stronger associations with bacterial adhesion. Effect size (*β*, *r*) and cluster‐robust *p*‐values were evaluated. The full regression results are provided in the Supporting Information (see S2).

**FIGURE 17:**
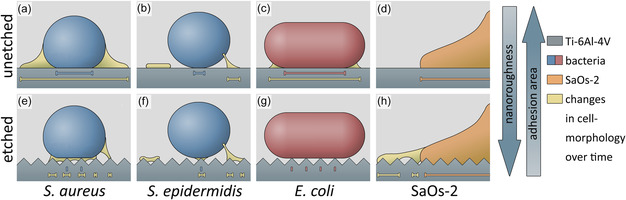
Schematic representation of cell–surface interactions on Ti–6Al–4V surfaces. The panels are arranged by cell type (columns) and surface morphology (rows), showing *S. aureus*, *S. epidermidis*, *E. coli*, and SaOs‐2 on unetched (a–d) and etched (e–h) samples. The drawings illustrate differences in cell geometry and contact area as observed across the different surface conditions by fluorescence and SEM imaging.

Because only a limited number of discrete roughness conditions was investigated, this correlation analysis should be interpreted as an exploratory trend analysis rather than as confirmatory evidence for direct causal relationships. The influence of topography was generally more pronounced at 1 h, indicating that early‐stage adhesion is predominantly governed by physical surface architecture. At 24 h, correlations weakened or became more strain‐specific, suggesting increasing biological adaptation over time. The regression screening also revealed that parameters reflecting horizontal distribution of nanoscale features (PtP, peak density) showed the most consistent associations with bacterial covered area, whereas classical roughness descriptors such as *R*_a_, *R*_q_, and *R*_sk_ displayed slightly weaker and sometimes strain‐dependent relationships. Overall, the data suggest that nanocontact points are among the relevant topographical factors influencing bacterial adhesion. Together with the SEM observations, these OLS‐supported trends provide the basis for the proposed mechanistic model summarized in Figure 17. This schematic illustrates the corresponding contact geometries and early morphological cell adaptations on unetched and nanostructured Ti–6Al–4V surfaces.

All three bacterial species established continuous cell–sample interfaces on the unetched samples (Figure 17a–c). In analogy to classical wetting models derived from WCA measurements, these interactions correspond to a Wenzel‐like wetting state in which the bacterial envelope closely follows the underlying topography. In contrast, the nanostructured surface (Figure 17e–‍g) induces a pronounced geometrical decoupling between cell envelope and sample surface. The lateral peak density remained nearly constant during etching, while vertical roughness parameters (PtV, *R*_sa_) increased substantially. Therefore, bacterial cells primarily contacted the surface at discrete nanoscale maxima while spanning the intervening valleys, resembling a Cassie–Baxter‐like contact geometry (see Figures 10–12). This interpretation is supported by the OLS regression screening, which identified PtP and peak density as the topographical parameters most consistently associated with bacterial covered area. Consequently, further increases in vertical roughness (PtV, *R*_sa_) are expected to produce only limited additional reductions in effective contact after the initial lateral reorganization of nanoscale maxima. This is consistent with our biological data, which show that the major adhesion change coincided with the initial formation of laterally distributed nanoscale maxima, whereas subsequent predominantly vertical deepening has a smaller incremental effect. In line with nanoscale contact mechanics concepts described by Dewald et al. and Dauben et al., adhesion can thus be viewed as confined to a limited number of discrete nanoscopic contact points, with their lateral distribution acting as the primary determinant of stable bacterial attachment [35, 49].

The more pronounced adhesion‐reducing effect observed here, relative to the results of Lüdecke et al., likely arises from differences in surface roughness and experimental design. Their study focused on low‐amplitude nanotopographies and early time points, making a direct quantitative comparison inappropriate, but the qualitative trend is consistent [31].

*S. aureus* appeared slightly flattened, generating a larger effective contact area. After 24 h, *S. aureus* exhibited a pronounced skirt‐like reshaping at the cell–substrate interface, progressively reducing the void spaces between the bacterial envelope and the sample surface. *S. epidermidis* remained more spherical and mechanically stiffer than *S. aureus*, consistent with the limited deformation observed in Figure 11. This reduced contact interface may concentrate the adhesive load onto a small number of nanoscale contact points, increasing the mechanical stress per bond and potentially rendering early attachment mechanically fragile. The SaOs‐2 cells (Figure 17d,h) adhered robustly on both unetched and nanostructured surfaces, forming noticeably extended lamellipodia that bridged across the nanoscale pores and reflecting enhanced cytoskeletal engagement with the underlying topography (see Figure 14).

Collectively, these observations support a physical adhesion model in which NaOH‐etched Ti–6Al–4V surfaces exhibit progressively increasing vertical relief. This geometry reduces the real contact area and the number of available nanoadhesion points for bacteria, thereby weakening early‐stage attachment, while SaOs‐2 cells with larger contact footprints and active cytoskeletal remodeling can overcome and integrate the same nanotopography. This mechanistic separation explains the selective suppression of bacterial adhesion on nanostructured titanium while maintaining compatibility with osteoblasts.

## 4 Conclusions

This study demonstrated that alkaline etching of Ti–6Al–4V produced nanostructured surfaces with increased roughness and hydrophilicity. These surface modifications altered early bacterial adhesion across three clinically relevant bacterial strains, with significant reductions observed for specific species and time points. At the same time, osteoblast‐like cells remained compatible with the nanostructured surfaces, indicating that reduced bacterial adhesion was achieved without compromising mammalian cell response.

The observed reduction in early bacterial adhesion, together with maintained osteoblast compatibility, suggests that nanoscale surface modification of Ti–6Al–4V is a promising complementary approach for the development of infection‐reducing orthopedic implant surfaces. However, these findings are an initial in vitro indication and further studies using more complex biological systems are required to confirm these findings. Future investigations should include protein‐containing environment, longer observation periods, polymicrobial conditions, biofilm models, immune‐cell interactions, and in vivo studies to evaluate the antibacterial and biological performance of these nanostructured surfaces under physiologically relevant conditions.

## Funding

This study was supported by the Deutsche Forschungsgemeinschaft (DFG, German Research Foundation) – 444711651, RTG 2723 Materials–Microbes–Microenvironments (M‐M‐M).

## Conflicts of Interest

The authors declare no conflicts of interest.

## Supporting information

Supplementary Material

## Data Availability

The raw data that support the findings of this study are available from the corresponding author upon reasonable request.

## References

[1] M. Geetha , A. K. Singh , R. Asokamani , and A. K. Gogia , “Ti Based Biomaterials, the Ultimate Choice for Orthopaedic Implants–A Review,” Progress in Materials Science 54 (2009): 397–425, 10.1016/j.pmatsci.2008.06.004.

[2] E. Marin and A. Lanzutti , “Biomedical Applications of Titanium Alloys: A Comprehensive Review,” Materials 17 (2024): 114, 10.3390/ma17010114. PMC10780041 38203968

[3] R. Pivec , A. J. Johnson , S. C. Mears , and M. A. Mont , “Hip Arthroplasty,” The Lancet 380 (2012): 1768–1777, 10.1016/S0140-6736(12)60607-2. 23021846

[4] A. J. Carr , O. Robertsson , S. Graves , et al., “Knee Replacement,” The Lancet 379 (2012): 1331–1340, 10.1016/S0140-6736(11)60752-6. 22398175

[5] A. R. MacLeod , M. Bishop , A. C. Longo , A. Shokrani , C. R. Bowen , and H. S. Gill , “Additively Produced Ti‐6Al‐4V Osteosynthesis Devices Meet the Requirements for Tensile Strength and Fatigue,” Journal of Manufacturing and Materials Processing 9 (2025): 227, 10.3390/jmmp9070227.

[6] G. Kacholi and M. G. Anasel , Health at a Glance 2023: OECD Indicators (OECD Publishing, 2023).

[7] C. Pabinger , H. Lothaller , N. Portner , and A. Geissler , “Projections of Hip Arthroplasty in OECD Countries up to 2050,” HIP International 28 (2018): 498–506, 10.1177/1120700018757940. 29783896

[8] A. J. Tande and R. Patel , “Prosthetic Joint Infection,” Clinical Microbiology Reviews 27 (2014): 302–345, 10.1128/CMR.00111-13. 24696437 PMC3993098

[9] T. A. Luthringer , Y. A. Fillingham , K. Okroj , E. J. Ward , and C. Della Valle , “Periprosthetic Joint Infection after Hip and Knee Arthroplasty: A Review for Emergency Care Providers,” Annals of Emergency Medicine 68 (2016): 324–334, 10.1016/j.annemergmed.2016.03.004. 27083857

[10] A. Premkumar , D. A. Kolin , K. X. Farley , et al., “Projected Economic Burden of Periprosthetic Joint Infection of the Hip and Knee in the United States,” The Journal of Arthroplasty 36 (2021): 1484–1489.e3, 10.1016/j.arth.2020.12.005. 33422392

[11] R. Patel , “Periprosthetic Joint Infection,” New England Journal of Medicine 388 (2023): 251–262, 10.1056/NEJMra2203477. 36652356

[12] D. G. J. Larsson and C.‐F. Flach , “Antibiotic Resistance in the Environment,” Nature Reviews Microbiology 20 (2022): 257–269, 10.1038/s41579-021-00649-x. 34737424 PMC8567979

[13] A. G. Nowotnick , Z. Xi , Z. Jin , et al., “Antimicrobial Biomaterials Based on Physical and Physicochemical Action,” Advanced Healthcare Materials 13 (2024): 2402001, 10.1002/adhm.202402001. 39301968 PMC11670291

[14] L. Tuchscherr , E. Medina , M. Hussain , et al., “Phenotype Switching: An Effective Bacterial Strategy to Escape Host Immune Response and Establish a Chronic Infection,” EMBO Molecular Medicine 3 (2011): 129–141, 10.1002/emmm.201000115 21268281 PMC3395110

[15] B. C. Kahl , K. Becker , and B. Löffler , “Clinical Significance and Pathogenesis of Staphylococcal Small Colony Variants in Persistent Infections,” Clinical Microbiology Reviews 29 (2016): 401–427, 10.1128/cmr.00069-15. 26960941 PMC4786882

[16] T. Albrektsson , P. I. Brånemark , H. A. Hansson , and J. Lindström , “Osseointegrated Titanium Implants: Requirements for Ensuring a Long‐Lasting, Direct Bone‐to‐Implant Anchorage in Man,” Acta Orthopaedica Scandinavica 52 (2009): 155–170, 10.3109/17453678108991776. 7246093

[17] I. Wall , N. Donos , K. Carlqvist , F. Jones , and P. Brett , “Modified Titanium Surfaces Promote Accelerated Osteogenic Differentiation of Mesenchymal Stromal Cells In Vitro,” Bone 45 (2009): 17–26, 10.1016/j.bone.2009.03.662. 19332166

[18] M. Pilia , T. Guda , S. M. Shiels , and M. R. Appleford , “Influence of Substrate Curvature on Osteoblast Orientation and Extracellular Matrix Deposition,” Journal of Biological Engineering 7 (2013): 23, 10.1186/1754-1611-7-23. 24090183 PMC3851034

[19] B. Geiger , J. P. Spatz , and A. D. Bershadsky , “Environmental Sensing through Focal Adhesions,” Nature Reviews Molecular Cell Biology 10 (2009): 21–33, 10.1038/nrm2593. 19197329

[20] T. Foster , “Staphylococcus,” in Medical Microbiology, 4th ed. (University of Texas Medical Branch, 1996). 21413338

[21] M. Katsikogianni and Y. F. Missirlis , “Concise Review of Mechanisms of Bacterial Adhesion to Biomaterials and of Techniques Used in Estimating Bacteria‐Material Interactions,” European Cells & Materials 8 (2004): 37–57, 10.22203/ecm.v008a05. 15593018

[22] A. G. Gristina , “Biomaterial‐Centered Infection: Microbial Adhesion versus Tissue Integration,” Science 237 (1987): 1588–1595, 10.1126/science.3629258. 3629258

[23] C. Mas‐Moruno , B. Su , and M. J. Dalby , “Multifunctional Coatings and Nanotopographies: Toward Cell Instructive and Antibacterial Implants,” Advanced Healthcare Materials 8 (2019): e1801103, 10.1002/adhm.201801103. 30468010

[24] P. R. L. Dabare , A. Bachhuka , J. Y. Quek , L. F. Marsal , J. Hayball , and K. Vasilev , “Nano‐Roughness‐Mediated Macrophage Polarization for Desired Host Immune Response,” Small Science 3 (2023): 2300080, 10.1002/smsc.202300080. 40213139 PMC11935885

[25] M. Jäger , C. Zilkens , K. Zanger , and R. Krauspe , “Significance of Nano‐and Microtopography for Cell‐Surface Interactions in Orthopaedic Implants,” BioMed Research International 2007 (2007): 069036, 10.1155/2007/69036. PMC2233875 18274618

[26] K. Wang , F. Rong , H. Peng , et al., “Infection Microenvironment‐Responsive Coating on Titanium Surfaces for On‐Demand Release of Therapeutic Gas and Antibiotic,” Advanced Healthcare Materials 13 (2024): e2304510, 10.1002/adhm.202304510. 38532711

[27] Q. Wang , Y. Gao , Y. Chen , et al., “Synergistic Enhancement of Antibacterial and Osteo‐Immunomodulatory Activities of Titanium Implants via Dual‐Responsive Multifunctional Surfaces,” Advanced Healthcare Materials 14 (2025): e2404260, 10.1002/adhm.202404260. 39690750

[28] E. P. Ivanova , J. Hasan , H. K. Webb , et al., “Bactericidal Activity of Black Silicon,” Nature Communications 4 (2013): 2838, 10.1038/ncomms3838. PMC3868328 24281410

[29] S. Pogodin , J. Hasan , V A. Baulin , et al., “Biophysical Model of Bacterial Cell Interactions with Nanopatterned Cicada Wing Surfaces,” Biophysical Journal 104 (2013): 835–840, 10.1016/j.bpj.2012.12.046. 23442962 PMC3576530

[30] D. P. Linklater , V. A. Baulin , S. Juodkazis , R. J. Crawford , P. Stoodley , and E. P. Ivanova , “Mechano‐Bactericidal Actions of Nanostructured Surfaces,” Nature Reviews Microbiology 19 (2021): 8–22, 10.1038/s41579-020-0414-z. 32807981

[31] C. Lüdecke , J. Bossert , M. Roth , and K. D. Jandt , “Physical Vapor Deposited Titanium Thin Films for Biomedical Applications: Reproducibility of Nanoscale Surface Roughness and Microbial Adhesion Properties,” Applied Surface Science 280 (2013): 578–589, 10.1016/j.apsusc.2013.05.030.

[32] R. Bright , A. Hayles , J. Wood , et al., “Bio‐Inspired Nanostructured Ti‐6Al‐4V Alloy: The Role of Two Alkaline Etchants and the Hydrothermal Processing Duration on Antibacterial Activity,” Nanomaterials 12 (2022): 1140, 10.3390/nano12071140. 35407257 PMC9000892

[33] J. V. Wandiyanto , V. K. Truong , M. Al Kobaisi , et al., “The Fate of Osteoblast‐Like MG‐63 Cells on Pre‐Infected Bactericidal Nanostructured Titanium Surfaces,” Materials 12 (2019): 1575, 10.3390/ma12101575. 31091694 PMC6567816

[34] X. Li , M. Qi , X. Sun , et al., “Surface Treatments on Titanium Implants via Nanostructured Ceria for Antibacterial and Anti‐Inflammatory Capabilities,” Acta Biomaterialia 94 (2019): 627–643, 10.1016/j.actbio.2019.06.023. 31212111

[35] C. Dewald , C. Lüdecke , I. Firkowska‐Boden , M. Roth , Jörg Bossert , and K. D. Jandt , “Gold Nanoparticle Contact Point Density Controls Microbial Adhesion on Gold Surfaces,” Colloid Surface B 163 (2018): 201–208, 10.1016/j.colsurfb.2017.12.037. 29304434

[36] S. Ferraris , A. Venturello , M. Miola , A. Cochis , L. Rimondini , and S. Spriano , “Antibacterial and Bioactive Nanostructured Titanium Surfaces for Bone Integration,” Applied Surface Science 311 (2014): 279–291, 10.1016/j.apsusc.2014.05.056.

[37] M. Kunrath , R. dos Santos , S. D. de Oliveira , R. Hubler , P. D. Sesterheim , and E. Teixeira , “Osteoblastic Cell Behavior and Early Bacterial Adhesion on Macro‐, Micro‐, and Nanostructured Titanium Surfaces for Biomedical Implant Applications,” The International Journal of Oral & Maxillofacial Implants 35 (2020): 773–781, 10.11607/jomi.8069. 32724931

[38] H. J. Moon , K. Gulati , T. Li , C. S. Moran , and S. Ivanovski , “Inflammatory or Reparative? Tuning Macrophage Polarization Using Anodized Anisotropic Nanoporous Titanium Implant Surfaces,” Small Science 4 (2024): 2400211, 10.1002/smsc.202400211. 40212258 PMC11935055

[39] M. F. Kunrath , G. Farina , L. B. S. Sturmer , and E. R. Teixeira , “TiO(2) Nanotubes as an Antibacterial Nanotextured Surface for Dental Implants: Systematic Review and Meta‐Analysis,” Dental Materials 40 (2024): 907–920, 10.1016/j.dental.2024.04.009. 38714394

[40] C. Pérez‐Jorge , A. Conde , M. A. Arenas , et al., “In Vitro Assessment of Staphylococcus Epidermidis and Staphylococcus Aureus Adhesion on TiO2 Nanotubes on Ti‐6Al‐4V Alloy,” Journal of Biomedical Materials Research Part A 100A (2012): 1696–1705, 10.1002/jbm.a.34118. 22447745

[41] B. Valdez‐Salas , E. Beltrán‐Partida , S. Castillo‐Uribe , et al., “In Vitro Assessment of Early Bacterial Activity on Micro/Nanostructured Ti6Al4V Surfaces,” Molecules 22 (2017): 832, 10.3390/molecules22050832. 28524087 PMC6154628

[42] D. Campoccia , L. Montanaro , and C. R. Arciola , “The Significance of Infection Related to Orthopedic Devices and Issues of Antibiotic Resistance,” Biomaterials 27 (2006): 2331–2339, 10.1016/j.biomaterials.2005.11.044. 16364434

[43] P. D. Fey and M. E. Olson , “Current Concepts in Biofilm Formation of Staphylococcus Epidermidis,” Future Microbiology 5 (2010): 917–933, 10.2217/Fmb.10.56. 20521936 PMC2903046

[44] R. Bright , D. Fernandes , J. Wood , et al., “Long‐Term Antibacterial Properties of a Nanostructured Titanium Alloy Surface: An In Vitro Study,” Materials Today Bio 13 (2022): 100176, 10.1016/j.mtbio.2021.100176. PMC8661698 34938990

[45] S. Komasa , Y. Taguchi , H. Nishida , M. Tanaka , and T. Kawazoe , “Bioactivity of Nanostructure on Titanium Surface Modified by Chemical Processing at Room Temperature,” Journal of Prosthodontic Research 56 (2012): 170–177, 10.1016/j.jpor.2011.12.002. 22613954

[46] S. Komasa , T. Kusumoto , Y. Taguchi , et al., “Effect of Nanosheet Surface Structure of Titanium Alloys on Cell Differentiation,” Journal of Nanomaterials 2014 (2014): 642527, 10.1155/2014/642527.

[47] P. Virtanen , R. Gommers , T. E. Oliphant , et al., “SciPy 1.0: Fundamental Algorithms for Scientific Computing in Python,” Nature Methods 17 (2020): 261–272, 10.1038/s41592-019-0686-2. 32015543 PMC7056644

[48] C. R. Harris , K. J. Millman , Séfan J. van der Walt , et al., “Array Programming with NumPy,” Nature 585 (2020): 357–362, 10.1038/s41586-020-2649-2. 32939066 PMC7759461

[49] T. J. Dauben , C. Dewald , I. Firkowska‐Boden , et al., “Quantifying the Relationship between Surfaces’ Nano‐Contact Point Density and Adhesion Force of Candida Albicans,” Colloid Surface B 194 (2020): 111177, 10.1016/j.colsurfb.2020.111177. 32569885

[50] Y. Luo , Y. Jiang , J. Zhu , J. Tu , and S. Jiao , “Surface Treatment Functionalization of Sodium Hydroxide onto 3D Printed Porous Ti6Al4V for Improved Biological Activities and Osteogenic Potencies,” Journal of Materials Research and Technology 9 (2020): 13661–13670, 10.1016/j.jmrt.2020.09.076.

[51] P. Thomsen , C. Larsson , L. E. Ericson , L. Sennerby , J. Lausmaa , and B. Kasemo , “Structure of the Interface between Rabbit Cortical Bone and Implants of Gold, Zirconium and Titanium,” Journal of Materials Science: Materials in Medicine 8 (1997): 653–665, 10.1023/a:1018579605426. 15348816

[52] Y. T. Sul , C. B. Johansson , Y. Jeong , K. Röser , A. Wennerberg , and T. Albrektsson , “Oxidized Implants and Their Influence on the Bone Response,” Journal of Materials Science: Materials in Medicine 12 (2001): 1025–1031, 10.1023/a:1012837905910. 15348359

[53] E. Gemelli and N. H. A. Camargo , “Oxidation Kinetics of Commercially Pure Titanium,” Matéria (Rio De Janeiro) 12 (2007): 525–531, 10.1590/S1517-70762007000300014.

[54] B. Feng , J. Weng , B. C. Yang , et al., “Surface Characterization of Titanium and Adsorption of Bovine Serum Albumin,” Materials Characterization 49 (2002): 129–137, 10.1016/S1044-5803(02)00341-8.

[55] K. Ma , R. Zhang , J. Sun , and C. Liu , “Oxidation Mechanism of Biomedical Titanium Alloy Surface and Experiment,” International Journal of Corrosion 2020 (2020): 1678615, 10.1155/bp-linebreak2020/1678615.

[56] O. Durante , C. Di Giorgio , V. Granata , et al., “Emergence and Evolution of Crystallization in TiO Thin Films: A Structural and Morphological Study,” Nanomaterials 11 (2021): 1409, 10.3390/nano11061409. 34073645 PMC8227354

[57] C. W. Dannatt and H. J. T. Ellingham , “Roasting and Reduction Processes—a General Survey,” Discussions of the Faraday Society 4 (1948): 126–139, 10.1039/DF9480400126.

[58] C. Leyens , “Oxidation and Protection of Titanium Alloys and Titanium Aluminides,” in Titanium and Titanium Alloys: Fundamentals and Applications (Wiley, 2003).

[59] A. F. Carley , P. R. Chalker , J. C. Riviere , and M. W. Roberts , “The Identification and Characterisation of Mixed Oxidation States at Oxidised Titanium Surfaces by Analysis of X‐Ray Photoelectron Spectra,” Journal of the Chemical Society, Faraday Transactions 1: Physical Chemistry in Condensed Phases 83 (1987): 351–370, 10.1039/F19878300351.

[60] K. A. Kanaya and S. Okayama , “Penetration and Energy‐Loss Theory of Electrons in Solid Targets,” Journal of Physics D: Applied Physics 5 (1972): 43, 10.1088/0022-3727/5/1/308.

[61] D. Drouin , A. R.éal Couture , D. Joly , X. Tastet , V. Aimez , and R. Gauvin , “CASINO V2.42: A Fast and Easy‐to‐use Modeling Tool for Scanning Electron Microscopy and Microanalysis Users,” Scanning 29 (2007): 92–101, 10.1002/sca.20000. 17455283

[62] M. Atapour , A. Pilchak , G. S. Frankel , J. C. Williams , M. H. Fathi , and M. Shamanian , “Corrosion Behavior of Ti‐6Al‐4V with Different Thermomechanical Treatments and Microstructures,” Corrosion 66 (2010): 065004, 10.5006/1.3452400.

[63] C. Gaona‐Tiburcio , J. M. Jáquez‐Muñoz , D. Nieves‐Mendoza , et al., “Corrosion Behavior of Titanium Alloys (Ti CP2, Ti‐6Al‐2Sn‐4Zr‐2Mo, Ti‐6Al‐4V and Ti Beta‐C) with Anodized and Exposed in NaCl and H2SO4 Solutions,” Metals 14 (2024 ): 160, 10.3390/met14020160.

[64] J. D. Patel , M. Ebert , R. Ward , and J. M. Anderson , “Biofilm Formation: Effects of Biomaterial Surface Chemistry and Serum Proteins,” Journal of Biomedical Materials Research Part A 80A (2007): 742–751, 10.1002/jbm.a.31103. 17177270

[65] B. Wieland , G. Gunaratnam , L. Pätzold , et al., “Assessment of the Biofilm Formation Capacities of Staphylococcus Aureus Strains Newman and Newman D2C In Vitro and In Vivo,” Scientific Reports 15 (2025), 10.1038/s41598-025-00521-5. PMC12062259 40341159

[66] H. Büttner , D. Mack , and H. Rohde , “Structural Basis of Staphylococcus Epidermidis Biofilm Formation: Mechanisms and Molecular Interactions,” Frontiers in Cellular and Infection Microbiology 5 (2015), 10.3389/fcimb.2015.00014. PMC4330918 25741476

