# Supplementary material for: Nanostructured Ti–6Al–4V Reduces Adhesion of Several Bacterial Species: An In Vitro Study
Source: Small Sci. 2026 Jul 28;6(8):e70348. doi: 10.1002/smsc.70348 (PMC13431757; doi:10.1002/smsc.70348)
Supplement: Supplementary file 1 — Supplementary Material [file SMSC-6-e70348-s001.pdf]

Supplementary Information

S1. Representative fluorescence images and additional GFP control data

Representative fluorescence images are provided to document the visual appearance of bacterial surface coverage across all investigated bacterial strains, incubation times, and sample groups. In addition to SYTO 9-stained *S. aureus*, *S. epidermidis*, and *E. coli*, a GFP-expressing *S. aureus* strain was included as a fluorescence-based viability control. The GFP strain was primarily intended to confirm the presence of viable adherent *S. aureus* after short-term adhesion and was therefore included in the main manuscript only for the 1 h time point.

After 24 h, the GFP-expressing *S. aureus* emitted markedly less fluorescence signal despite unchanged imaging parameters. As a result, weakly fluorescent bacteria could fall below the segmentation threshold, and the quantified covered area may underestimate the actual bacterial surface coverage. Importantly, the same threshold and analysis conditions were retained to avoid introducing a time-point-specific evaluation bias. Therefore, the 24 h GFP data should only be interpreted within the GFP 24 h dataset itself and are not directly comparable to the 1 h GFP data or to the SYTO 9-stained datasets. For transparency, the representative 24 h GFP images and the corresponding quantitative evaluation are provided here as supplementary control data, but they were not used as a central endpoint in the main manuscript.

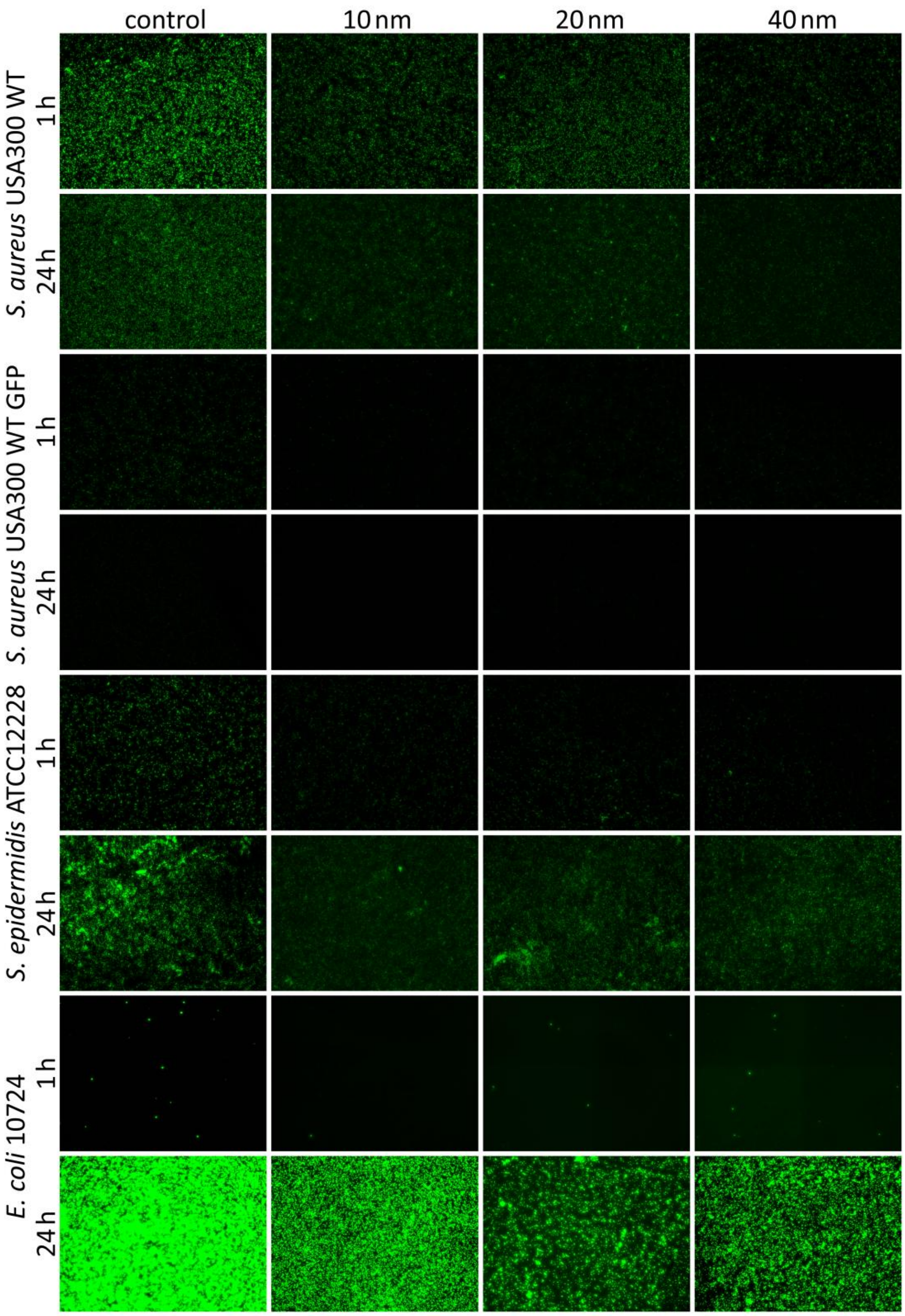

Figure S1: Images show *S. aureus*, *S.aureus* GFP, *S. epidermidis*, and *E. coli* after adhesion times of 1 h and 24 h on surfaces of the four sample groups respectivly. Green fluorescence indicates the detected bacterial signal. Images are shown as representative examples for qualitative comparison across bacterial strains, time points, and roughness groups. The reduced GFP emission after 24 h should be considered when interpreting the corresponding GFP images.

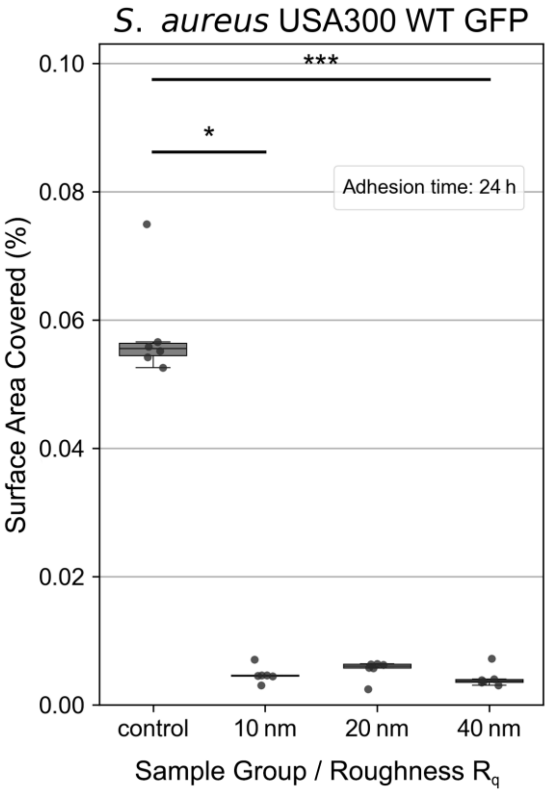

Figure S2: Boxplots showing surface area covered (%) of GFP expressing *S. aureus* USA300 WT on etched Ti-6Al-4V surfaces with nominal roughness values of 0.2 nm (control), 10 nm, 20 nm, and 40 nm after adhesion time of 24 h. Boxplots display medians and interquartile ranges; whiskers represent 1.5 × IQR; individual points correspond to per-sample values. Statistically significant differences between roughness groups were determined using the Kruskal–Wallis test ($\alpha = 0.05$) followed by Dunn's post-hoc test with Holm–Šidák correction. Significance levels: $p < 0.05$ (*), $p < 0.001$ (***).

S2. Linear regression screening of AFM-derived surface parameters

To systematically evaluate the association between nanoscale surface descriptors and bacterial surface coverage, separate univariate ordinary least squares (OLS) regressions were performed for each bacterial strain and adhesion time point. The dependent variable was Covered Area (%), and each AFM-derived parameter (R_a, R_q, R_{sk}, R_{sa}, peak density, PtP,

PtV) was tested individually as independent variable. Cluster-robust standard errors were applied using roughness group as clustering variable to account for non-independence of image-level measurements within identical surface conditions. For each model, β, R², r = sign(β)·√R², and the clustered p-value were extracted.

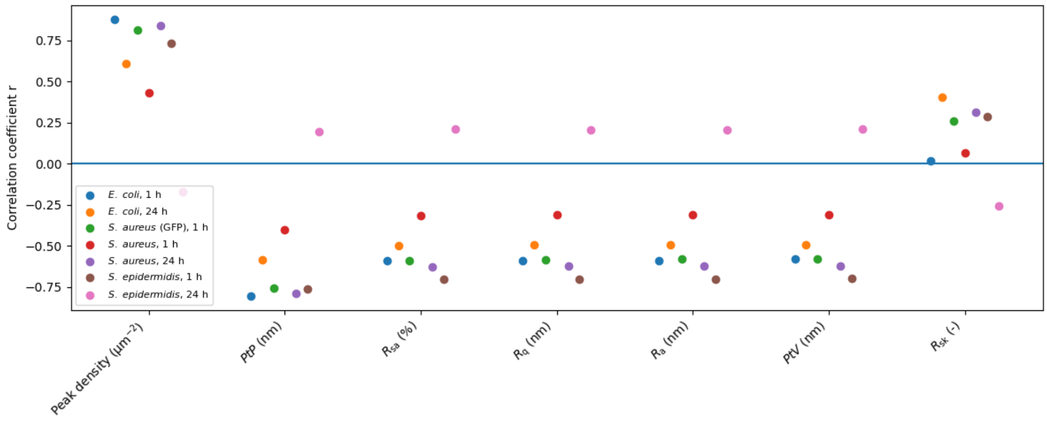

Figure S3: Correlation coefficients (r) for univariate OLS regressions between AFM-derived surface parameters and bacterial covered area. Each point represents one strain–time combination. The horizontal line indicates r = 0. Parameters are ordered according to mean(|r|).

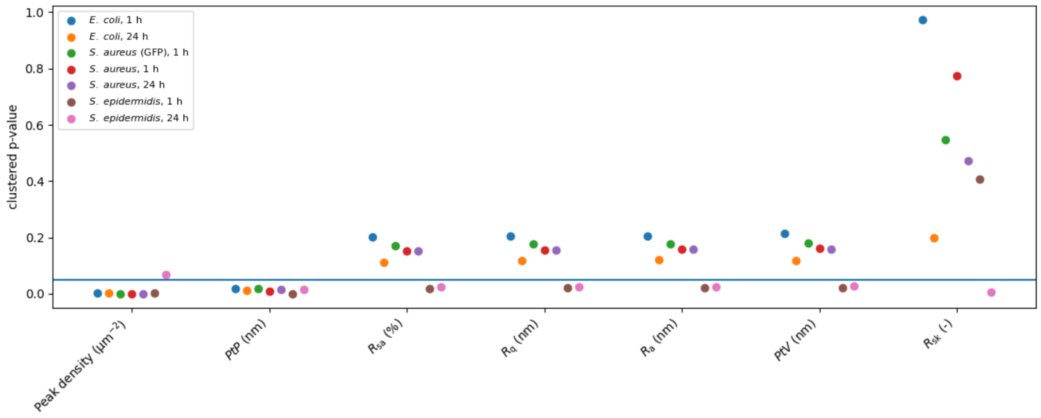

Figure S4: Cluster-robust p-values for the slope coefficient (β_1) of the univariate OLS regressions between AFM-derived surface parameters and bacterial covered area. The p-values correspond to the hypothesis test H_0: $\beta_1 = 0$ (no linear association). Each point represents one strain–time combination (1 h, 24 h). The blue horizontal line indicates $\alpha = 0.05$.
